# Detection of ongoing asymptomatic *Porcine deltacoronavirus* infections via transiently produced IgGs using protein-peptide hybrid microarray

**DOI:** 10.3389/fmicb.2026.1846662

**Published:** 2026-06-24

**Authors:** Mengyu Li, Jizong Li, Lan Yang, Zhiwei Li, Dayong Gu, Xiaoting Xu, Yezi Liu, Xiaoyu Li, Wei Wang, Bin Li, Hongwei Ma

**Affiliations:** 1 School of Nano-Tech and Nano-Bionics, University of Science and Technology of China, Hefei, China; 2 Division of Nanobiomedicine, Suzhou Institute of Nano-Tech and Nano-Bionics, Chinese Academy of Sciences, Suzhou, China; 3 Institute of Veterinary Medicine, Jiangsu Academy of Agricultural Sciences, Key Laboratory of Veterinary Biological Engineering and Technology Ministry of Agriculture, Jiangsu Key Laboratory for Food Quality and Safety-State Key Laboratory Cultivation Base of Ministry of Science and Technology, Nanjing, China; 4 Clinical Laboratory Center, People's Hospital of Xinjiang Uygur Autonomous Region, Urumqi, Xinjiang, China; 5 Department of Laboratory Medicine, Shenzhen Institute of Translational Medicine, The First Affiliated Hospital of Shenzhen University, Shenzhen Second People's Hospital, Medical Innovation Technology Transformation Center of Shenzhen Second People's Hospital, Shenzhen University, Shenzhen, China; 6 Department of Radiation Oncology, The First Affiliated Hospital of Soochow University, Suzhou, China

**Keywords:** asymptomatic infections, immunogenicity, PDCoV, transiently produced IgGs, zoonotic

## Abstract

**Background:**

Asymptomatic hosts can shed pathogens without showing clinical symptoms, making them invisible to routine screenings and potent drivers of pathogen dissemination and epidemic outbreaks. The lack of reliable, cost-effective tools for large-scale identification of asymptomatic infections hampers early intervention and control strategies. *Porcine deltacoronavirus* (PDCoV), a zoonotic pathogen with potential for cross-species transmission, presents a critical case for improving such detection methodologies.

**Methods:**

We improved the IgG serodynamics-based epitope discovery method by integrating clustering and high-level analysis, which helped us identify linear epitopes with immunogenicity from a large number of candidate epitopes. Epitopes were filtered using negative sera identified by virus neutralization tests (VNT) to eliminate highly antigenic probes. These remaining low antigenicity probes were used to construct a protein-peptide hybrid microarray (PPHM_PDCoV_). The platform was applied to detect PDCoV-specific transiently produced IgGs (TPIs) in serum samples collected from pigs aged 28–174 days.

**Results:**

The PPHM_PDCoV_ successfully detected asymptomatic PDCoV infections in pigs, particularly showing a peak infection rate of 15% at 45 days of age. The platform enabled not only the detection of asymptomatic carriers but also the characterization of infection stages.

**Conclusion:**

The study provides a novel, specific, and practical platform for detecting asymptomatic PDCoV infections based on serological TPI signatures. It offers early warning and disease prevention strategies in livestock and establishes a framework for future monitoring of potential interspecies transmission.

## Graphical Abstract

**Figure:**
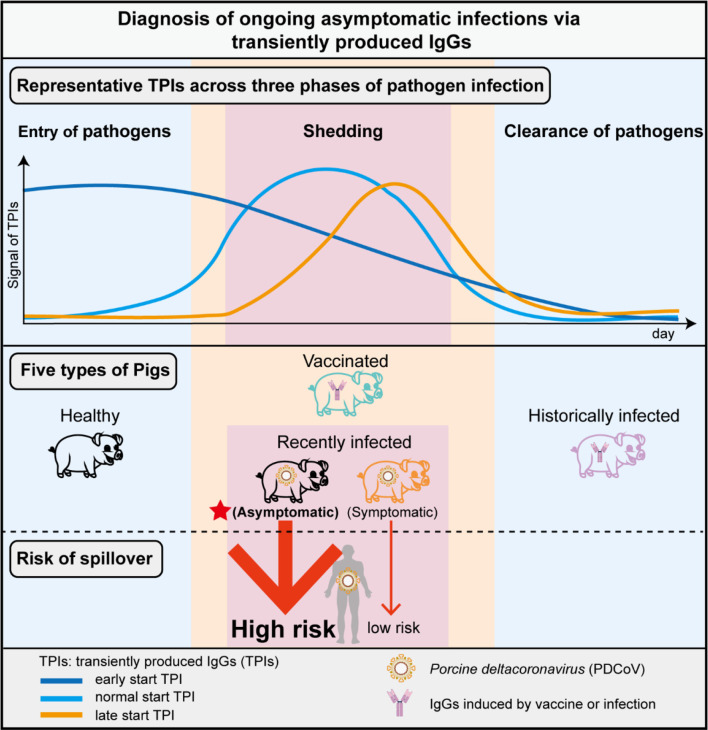

## Introduction

Zoonosis constitute 75% of emerging infectious diseases, presenting significant challenges to public health (Taylor et al., 2001). Since 2003, zoonotic coronaviruses have caused at least three major epidemic events, including the early *Severe Acute Respiratory Syndrome* (SARS), *Middle East Respiratory Syndrome* (MERS), and the 2019 pandemic of *Severe Acute Respiratory Syndrome Coronavirus 2* (SARS-CoV-2) (Su et al., 2016; Wu et al., 2020). To date, SARS-CoV-2 persists asymptomatically in various tissues of the body over the long term (Zuo et al., 2024). In 2012, a novel coronavirus *Porcine deltacoronavirus* (PDCoV) was first reported in Hong Kong, China (Woo et al., 2012; Wang et al., 2014). Experimental evidence suggests that PDCoV can enter cells of pigs, humans and poultry through specific receptors (Li et al., 2018; Ji et al., 2022). It also has been demonstrated to infect chickens (Liang et al., 2019), calves (Jung et al., 2017), and mice (Liu et al., 2021) in laboratory. PDCoV not only exists in livestock but also impacts humans as an emerging coronavirus. In 2021, three children in Haiti were reported to have been infected (Lednicky et al., 2021), posing a threat to the public health security.

PDCoV can cause severe atrophic enteritis in piglets accompanied by diarrhea, vomiting and dehydration. The mortality rate of suckling piglets is as high as 40%−80%, resulting in huge losses (Kong et al., 2022). However, the harm to adult pigs is not significant, which is very likely to change into asymptomatic infections (Yen et al., 2022). Asymptomatic infections are difficult to detect because they do not have any symptoms, but they are also infectious, increasing the risk of covert transmission of the epidemic (Gandhi et al., 2020). With an annual pig population of 700 million in China, widespread asymptomatic infections could lead to PDCoV mutations and spillover, similar to the mutation of *porcine circovirus 2* (PCV2) (Karuppannan and Opriessnig, 2017).

Traditional detection methods for asymptomatic infections include viral DNA, antigen, or antibody detection, but these approaches have several limitations: (1) Window Period: the polymerase chain reaction (PCR) is due to the DNA of virus may not be detected in the early phase of infection, requiring time for the viral DNA to reach detectable levels (Boum et al., 2021). The antigen or antibody detection is determined by the time needed for the human immune system to respond to the newly invading virus, as well as the sensitivity and specificity of the enzyme-linked immunosorbent assay (ELISA) detection technology (Liu and Rusling, 2021). (2) Unable to Determine the Phase of Infection: PCR is inadequate for distinguishing between recent and historical infections, while ELISA require the collection of samples at multiple time to observe the trends of IgG and IgM. The method is time-consuming and can yield ambiguous results (Varghese et al., 2018). (3) Incapable in Differentiating Infected from Vaccinated Animals (DIVA; *Specialized abbreviations used throughout the manuscript are defined in* *Supplementary Table S1*): vaccines are an effective means of infection protection. After emergency immunization, identifying and disposing of infected animals is key to stopping the spread of the epidemic. But traditional methods of DIVA rely on the development of “marker vaccines” (Meeusen et al., 2007; Gómez-Sebastián et al., 2008). Therefore, there is an urgent need to develop a new method for monitoring asymptomatic infections to determine the true prevalence of PDCoV.

We have demonstrated the application of viral peptides (*i.e*., linear epitopes) in pathogen detection, DIVA, and vaccine efficacy evaluation (Chen et al., 2022; Xue et al., 2020). In addition, we developed Immunoglobuin G (IgG) sero-dynamics (IsD) curves aided epitope discovery (IsDAED) method, using PCV2 of simple structure as the model virus. The method classifies probes into three patterns—specific interaction (SI), non-specific interaction (NSI), and null interaction (Null)—based on their distinct IgG sero-dynamics (IsD) curves. Although the method enables accurate identification of linear epitopes, its universal application still faces three challenges: (1) Applicability in Multi-protein Pathogens: while IsD curves have been successfully used for PCV2, which has only one major structural protein (*i.e*., Cap), their applicability to multi-protein pathogens has not been explored. (2) Optimization of Diagnostic Probes: in simple pathogens with few candidate probes, direct use of probes like the five linear epitopes from PCV2 is possible (Chen et al., 2022). However, when the pathogen is structurally complex and has multi proteins, selecting the optimal diagnostic epitopes from a larger number of candidate probes presents a new challenge. (3) Limited Ability to Judge Antigenicity: the IsDAED method is known for determining the immunogenicity of probes. Although it can exclude some non-specific interaction (NSI) probes, the longitudinal cohort used by IsDEAD provides limited antibody diversity, failing to reflect real world serum samples. To date, research on PDCoV epitope has largely focused on proteins, with few studies on peptide epitopes.

In the study, pigs challenged with PDCoV were selected to obtain high frequency longitudinal cohort samples. Using the “improved IsDAED” method, which integrates “clustering analysis” and “high-level analysis” to rapidly screen and prioritize optimal candidates from a large number of potential epitopes, we successfully identified PDCoV diagnostic epitopes that detect transiently produced IgGs (TPIs). Subsequently, negative sera determined by virus neutralization tests (VNT) were used to exclude highly antigenic probes. Probes with a response rate exceeding 20% in negative sera were removed, and the remaining qualified probes were assembled to generate the PDCoV protein-peptide hybrid microarray (PPHM_PDCoV_). Finally, using the PPHM_PDCoV_, we assessed asymptomatic PDCoV infections in pigs from non-endemic areas in China.

## Materials and methods

### Protein and peptide library

Protein S and N from the PDCoV strain CZ2020 (GenBank accession number: OK546242) was expressed by *baculovirus* and *Escherichia coli*, respectively (Li et al., 2023; Wang et al., 2021). The peptides come from the S (GenBank accession no. UDM84934.1), E (GenBank accession no. UDM84935.1), M (GenBank accession no. UDM84936.1), and N (GenBank accession no. UDM84938.1) proteins of PDCoV strain CZ2020, and proteins were employed to analyze the amino acid (aa) sequences. Twenty-mer peptides with an overlap of 10-aa residues covering the entire protein were chemically synthesized by Genescript Biochem (Nanjing, China), which finally yielded 150 peptides in total (Supplementary Figure S1), including 92 peptides from S (named S1 to S115), 6 from E (named E1 to E8), 19 from M (named M1 to M21), and 33 from N (named N1 to N34), respectively. 13 peptides derived from the PDCoV S protein (S60/61/68/69/72/75/76/78/87/90/91/108/109) were not synthesized. These peptides share more than 60% sequence homology with corresponding regions of the *porcine epidemic diarrhea virus* (PEDV) S protein and showed no seroreactivity in previous PEDV studies. A total of 15 peptides (S1/25/26/37/49/59/74/84/112/113 from S protein; E2 and E3 from E protein; M2 and M8 from M protein; N26 from N protein) failed final synthesis due to high hydrophobicity (grand average of hydropathicity, GRAVY > 0.5) or low purity (< 75%; Table 1).

**Table 1:** Summary of peptide synthesis for PDCoV structural proteins.

Protein	Amino acid position range	Planned number of peptides	Not synthesized number of peptides	Failed number of peptides	Obtained number of peptides
S	1–1,159	115	13	10	92
E	1–83	8	/	2	6
M	1–217	21	/	2	19
N	1–342	34	/	1	33
Total	/	178	/	15	150

### Protein-peptide hybrid microarray (PPHM)

The serological screening was performed using three successive generations of the PPHM platform, designated PPHM-#1, PPHM-#2, and PPHM_PDCoV_, each with a distinct probe composition as detailed below.

PPHM-#1 was composed of two sub-arrays, PPHM-#1-1 and PPHM-#1-2. PPHM-#1-1 contained a total of 134 detection peptides: 6 derived from the E protein, 19 from the M protein, 33 from the N protein, and 76 from the S protein (Supplementary Figure S2a). PPHM-#1-2 contained the remaining 16 peptides from the S protein, plus the full-length recombinant N and S proteins as additional probes (Supplementary Figure S2b). All probes were printed onto activated nano-membranes using a non-contact spotter (sciFLEXARRAYER S1, Scienion, Berlin, Germany). The peptides were diluted in a printing buffer consisting of 0.3 M PB, 0.2% glycerol, 0.01% Triton X-100, and 1.5% mannitol to a final concentration of 100 μg/ml, while the full-length N and S proteins were diluted in the same buffer and printed at 50 μg/ml. To control for assay performance, PPHM-#1-1 also included seven positive control spots printed with pig IgG at concentrations of 2, 4, 6, 8, 10, 12, and 14 μg/ml (diluted in the aforementioned printing buffer), and a negative control printed with only printing buffer (0.3 M PB, 0.2% glycerol, 0.01% Triton X-100, 1.5% mannitol). PPHM-#1-2 also contained positive/negative controls, with pig IgG (50 μg/ml, diluted in the printing buffer) as positive controls and the same printing buffer as negative controls.

PPHM-#2 was a simplified version of the platform (Supplementary Figure S2c). The probes included the full-length S and N proteins, 20 selected diagnostic peptides (M17/21, N5/8/9/10/19/29/30/33, S4/15/28/38/65/80/86/89/102/114), pig IgG as positive controls, and the printing buffer (0.3 M PB, 0.2% glycerol, 0.01% Triton X-100, 1.5% mannitol) as negative controls. All probes were printed using the same sciFLEXARRAYER S1 instrument. Peptides were diluted in the printing buffer to 100 μg/ml, while the full-length proteins and pig IgG were diluted in the same buffer and printed at 50 μg/ml.

The final diagnostic platform, PPHM_PDCoV_, contained only the most specific probes: 12 peptides (M17/21, N5/8/29, S4/15/28/38/65/80/102), pig IgG as positive controls, and the printing buffer (0.3 M PB, 0.2% glycerol, 0.01% Triton X-100, 1.5% mannitol) as negative controls (Supplementary Figure S2d). Printing conditions were consistent with previous arrays: peptides diluted in the printing buffer to 100 μg/ml and pig IgG (50 μg/ml, diluted in the printing buffer) using the sciFLEXARRAYER S1 spotter.

### Challenge and serum collection

**Figure 1:**
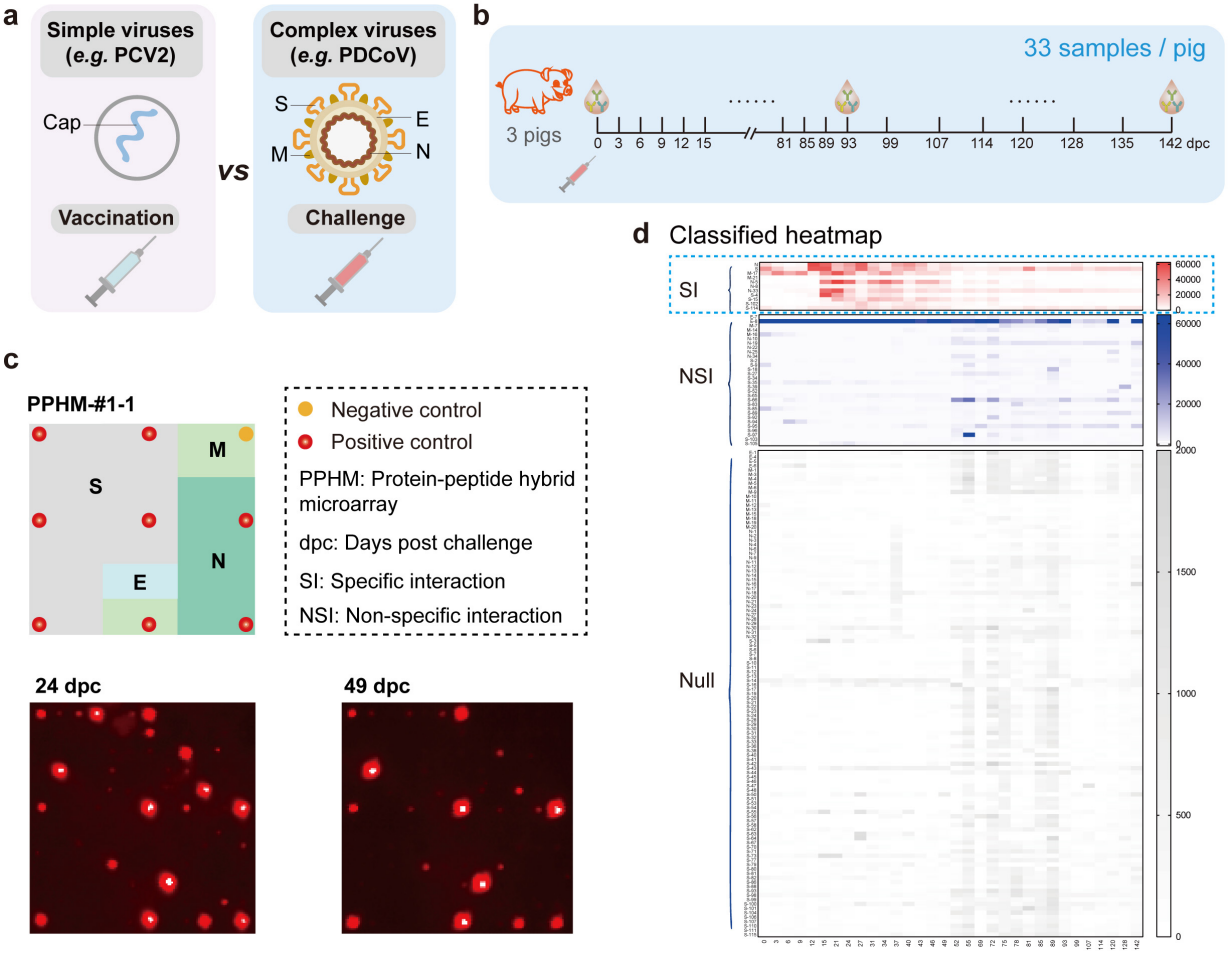
The IsDAED method reveals 20 candidate epitopes for PDCoV. **(a)** Structural proteins of simple and complex viruses, and methods of serum collection. Simple virus such as PCV2 have only one structural protein (Cap) and sera are obtained through immunization; while complex virus like PDCoV have four structural proteins (S, E, M, and N) and sera are obtained through challenge. **(b)** Experimental design of the PDCoV challenge group: three piglets were inoculated orally with PDCoV CZ2020 (10 ml × 10^6.0^ TCID_50_/ml per piglet) and collected the sera. **(c)** Composition of PPHM-#1-1 and screening results of pig1 at 24 dpc and 49 dpc. **(d)** Classified heatmap of pig1 probes (2 proteins and 150 peptides). They were divided into three types, 11 specific interaction (SI) probes (7.2%), 30 non-specific interaction (NSI) probes (19.7%), and 111 null probes (73%) respectively. High-resolution image is available in Supplementary Figure S1.

To induce a stronger immune response and gain more epitopes. Three piglets at 27 days of age were inoculated orally with PDCoV CZ2020 (10 ml × 10^6.0^ TCID_50_/ml per piglet) at 0 days post-challenge (dpc). Sera were taken from the three piglets at the intervals of 3–4 days (0–93 dpc), and 6–8 days after 93 dpc to 142 dpc, with 33 sera taken from each pig (Figure 1b).

### Vaccine

Two kinds of PDCoV vaccines, including an inactivated PDCoV vaccine and an S-based subunit vaccine, were used in the study. Both vaccines were described and validated in the previous published work by Li et al. (2023). The study aimed to compare IgG dynamic characteristics induced by PDCoV vaccination and viral challenge.

The inactivated vaccine contains whole inactivated PDCoV particles with complete viral antigenicity, whereas the S-based subunit vaccine is based on recombinant PDCoV spike (S) protein, which is the major immunogen capable of inducing specific protective antibodies. These two vaccines with distinct antigen compositions were applied to generate immune sera, providing fundamental samples for the development and evaluation of our diagnostic procedure.

### Inactivated vaccine immunization and serum collection

Nineteen piglets at 5-day-old were divided into two groups (group 1 with 5 pigs, group 2 with 14 pigs) and immunized intramuscularly the same dose of PDCoV inactivated vaccine (dose: 1 ml/10^7.5^ TCID_50_/ml per piglet) at 0 and 14 days (*i.e*., 0 days post-vaccination, dpv). The sera of 15 pigs in the inactivated vaccine group 1 was collected at 14 dpv, 24 dpv and 32 dpv, respectively. While group 2 only collected sera at 24 dpv.

### S-based subunit vaccine immunization and serum collection

In group 3, four piglets at 5-day-old were immunized intramuscularly the same dose of S subunit vaccine (2 ml × 200 μg per piglet) at 0 and 14 days. Serum samples were collected at 21 dpv. Group 4 consisted of five suckling pig from sows vaccinated with S-based protein vaccine at 40 and 20 days prior to parturition. Serum samples were collected from suckling piglets at 5 days after sow parturition.

### Challenge after vaccination and serum collection

To evaluate the protective effect of the PDCoV inactivated vaccine and compare the characteristics of anti-peptide IgG responses between vaccinated and unvaccinated pigs after viral challenge, we performed a vaccination-challenge experiment and sequentially collected serum samples for detection. In group 5 (experimental group), two 5-day-old piglets were intramuscularly immunized with the same dose of PDCoV inactivated vaccine (dose: 1 ml/10^7.5^ TCID_50_/ml per piglet) at 0 and 14 days. At 21 dpv, all piglets were orally challenged with PDCoV strain CZ2020 at a dose of 10 ml × 10^6.0^ TCID_50_/ml per piglet. Group 6 served as the blank control group without prior vaccination. Piglets in this group received the identical oral PDCoV CZ2020 challenge at the same time point and with the same viral dose as those in group 5. For both group 5 and group 6, serum samples were collected at 0, 7, 10, 13, 16, 19, 22, and 25 dpc.

### 50 PDCoV-negative sera

Fifty PDCoV-negative serum samples were collected to serve as negative controls for validating the antigenicity of our selected epitopes. Serum samples were collected from a pig farm in East China where PDCoV infection is not endemic. All animals were confirmed to be clinically healthy with no signs of respiratory or gastrointestinal diseases during a 2-week observation period. In addition to confirmation of negative anti-PDCoV antibody results by virus neutralization tests (VNT), all serum samples were further tested by PDCoV-specific RT-PCR, and all samples yielding negative PCR results. These tests confirmed the PDCoV-negative status of the samples, ensuring reliable antigenicity screening results.

### 311 human serum samples collection and grouping

We collected human serum samples to investigate potential PDCoV zoonotic transmission and assess viral exposure among human populations. A total of 311 serum samples were enrolled in the study. These samples were collected from multiple regions and different population groups to ensure representative serological screening data, including 3 samples from the farm workers at the Rudong Pig Farm in Jiangsu, 70 samples from the Clinical Laboratory Center of the Fourth Affiliated Hospital of Soochow University, 50 samples from the Clinical Laboratory Center of Shenzhen Second People's Hospital, and 188 samples from Clinical Laboratory Center of People's Hospital of Xinjiang Uygur Autonomous Region. Detailed information on the source and composition of all human serum samples is summarized in Supplementary Table S5.

### Serum collection and processing

All serum samples were collected and processed as follows: 3 ml of whole blood from the superior vena cava was (pig) or median cubital vein (human) collected with a disposable container and stationarily placed at 4 °C for 2 h; then the sample was centrifuged at 2,000 rpm for 15 min, and the supernatant was inactivated in a water bath at 56 °C for 30 min and finally stored at −70 °C.

### Serum screening with protein-peptide hybrid microarray

All longitudinal serum samples collected after challenge were first screened using PPHM-#1-1. Serum was first diluted 1:50 with serum dilution buffer (1% bovine serum albumin, 1% casein, 0.5% sucrose, 0.2% polyvinylpyrrolidone, 0.5% Tween 20 in 0.01 M phosphate-buffered saline, pH 7.4). Subsequently, 450 μl diluted serum was added into each microarray of PPHM-#1-1 and incubated for 30 min on a shaker (500 rpm, 37 °C). The microarray was then rinsed 3 times with washing buffer and incubated with 450 μl of horseradish peroxidase (HRP)-conjugated rabbit anti-pig IgG (Sigma-Aldrich) diluted 1:7,500 in peroxidase conjugate stabilizer/diluent (Thermo Scientific) for another 30 min on a shaker (500 rpm, 37 °C), followed by the same washing steps as described above. Added 100 μl of chemiluminescence substrate (Thermo Scientific) onto the microarray, and the images were taken at a wavelength of 635 nm using the Clear 4 imaging system (Suzhou Epitope, China). The signal of any peptide dot was defined as the signal readout of dot minus the signal readout of background. This incubation and washing procedure effectively reduced non-specific background signals and eliminated interference from non-neutralizing antibodies, improving the detection specificity and reliability of the microarray.

The experimental procedure of other samples in the study remained consistent with the protocol described above, except for the reaction volume: the volumes of serum and horseradish peroxidase (HRP)-conjugated rabbit anti-pig IgG were reduced from 450 μl to 100 μl, and the dosage of chemiluminescence substrate was decreased from 100 μl to 15 μl. When TMB-Blotting Solution was used instead, 80 μl of TMB chromogenic substrate (Thermo Scientific) was added to the microarray.

### qRT-PCR

Fresh fecal swabs were collected from piglets on a daily basis and immediately immersed in 1 ml of PBS. The viral RNA load in the fecal swabs was quantified via TaqMan-based real-time RT-PCR. In short, RNA was extracted from the supernatant of fecal and tissue samples following a previously described protocol (Gerdts and Zakhartchouk, 2017). The extracted RNA was then reverse transcribed into cDNA using the Vazyme Reverse Transcription Kit. The subsequent cDNA amplification was conducted using the Taq Pro HS Probe Master Mix (Vazyme, China) with the following primers: forward, 5′-ATCGACCACATGGCTCCAA-3′; reverse, 5′-CAGCTCTTGCCCATGTAGCTT-3′; and the probe, 5′-CACACCAGTCGTTAAGCATGGCAAGCT-3′ (Suzuki et al., 2018).

### Virus neutralization test

To evaluate neutralizing antibody titers, LLC-PK1 cells were seeded into 96-well culture plates (Thermo Fisher Scientific) at a density of 3 × 10^5^ cells/ml using 100 μl per well. The plates were then incubated at 37 °C in a humidified atmosphere of 5% CO_2_ overnight to achieve confluency of 80–90%. The collected serum samples were heat-inactivated at 56 °C incubated at 37 °C. Serial 2-fold dilutions of the sera replicates were prepared and mixed with an equivalent volume containing 100 TCID_50_ of PDCoV. These mixtures were incubated at 37 °C inactivation. Subsequently, each diluted serum was added to monolayers of LLC-PK1 cells in 96-well culture plates. After a 72 h incubation period, the cytopathic effect (CPE) was assessed. Neutralizing titers were quantified as the reciprocal of the highest serum dilution that resulted in complete neutralization of the virus.

### Statistical analysis

Microarray images acquired by the Clear 4 imaging system (Suzhou Epitope, China) were analyzed using GenePix software. The raw signal value of each spot was calculated as follows: signal intensity = peptide spot intensity – background intensity. A single probe was regarded as positive when the normalized signal value exceeded the filter value: >2,000 for the chemiluminescence substrate system, and >10 for the TMB chromogenic substrate system. Both filter values were derived from blank buffer signals measured on the PPHM platform following the formula: filter value = mean blank signal + 3 × standard deviation of blank buffer signal. Blank buffer samples collected during platform construction were used for this statistical calculation, and practical filter values were finally adjusted to 2,000 and 10 respectively for stricter background exclusion. For overall sample discrimination at the microarray level, a DMI cutoff value of 2 was applied to determine the positive or negative status of each serum sample. The DMI value was defined as the sum of binary probe results, in which each probe was assigned a value of 1 for positive response and 0 for negative response. Cluster analysis and heatmap visualization were performed using the “heatmap” package in R software. Hierarchical clustering was conducted based on Euclidean distance. Specifically, the dist() function was used to calculate Euclidean distance, and the hclust() function was applied for hierarchical clustering. All other statistical graphs were generated using GraphPad Prism software. All statistical procedures and cutoff thresholds were predefined and implemented consistently throughout the study to ensure reproducibility and reliability.

## Results

### IsDAED method is also suitable for accurately identifying PDCoV epitopes

In our previous work (Chen et al., 2022), we developed the IsDAED epitope identification method using PCV2 as a model virus. Here, we have applied the IsDAED method to a complex virus PDCoV with two significant improvements. In terms of viral composition, PDCoV is more complex than PCV2, which has only one structural protein (*i.e.*, Cap). The genome of PDCoV encodes four structural proteins: spike [S], envelope [E], membrane [M], and nucleocapsid [N]. Therefore, we expect that PDCoV will induce a more complex humoral immune response than PCV2 does. In terms of samples collected, we have employed “challenge” instead of “vaccination” used for PCV2 to obtain high-frequency longitudinal cohort sera of PDCoV (Figure 1a). The improvements can be attributed to two factors: (1) there are no commercial PDCoV vaccines available, and the two vaccines we developed did not induce TPIs, (see below); (2) based on past experience (Chen et al., 2022), longitudinal cohort samples of “challenge” can cover the full range of IgG sero-dynamics (IsD).

Following the approach, we selected three pigs that were confirmed clinically healthy and free of respiratory or gastrointestinal diseases after a 2-week observation period, and they were challenge at 27 days of age (*i.e.*, 0 days post-challenge, 0 dpc). Sera were collected at intervals of 3–4 days from 0 dpc to 93 dpc, and at intervals of 6–8 days from 93 dpc to 142 dpc, yielding a total of 33 sera per piglet (Figure 1b). In addition, fecal shedding of the virus was assessed in all three animals, providing further evidence of successful viral challenge (Supplementary Figure S3). Taking pig1 as an example, we screened the 33 sera with PPHM-#1 (consist of PPHM-#1-1 and PPHM-#1-2) to obtain the signal of responsive probes. PPHM-#1 consists of 150 20-mer peptides synthesized according the amino acid sequences of four structural proteins (*i.e.*, S, E, M and N), as well as two proteins: S and N (see materials and methods). As the dpc increased (24–49 dpc), the signal of responsive probes gradually disappeared (Figure 1c, Supplementary Figure S4). Notably, all serum samples from the three challenge pigs yielded negative results in quantitative real-time PCR (qRT-PCR) analysis.

**Figure 2:**
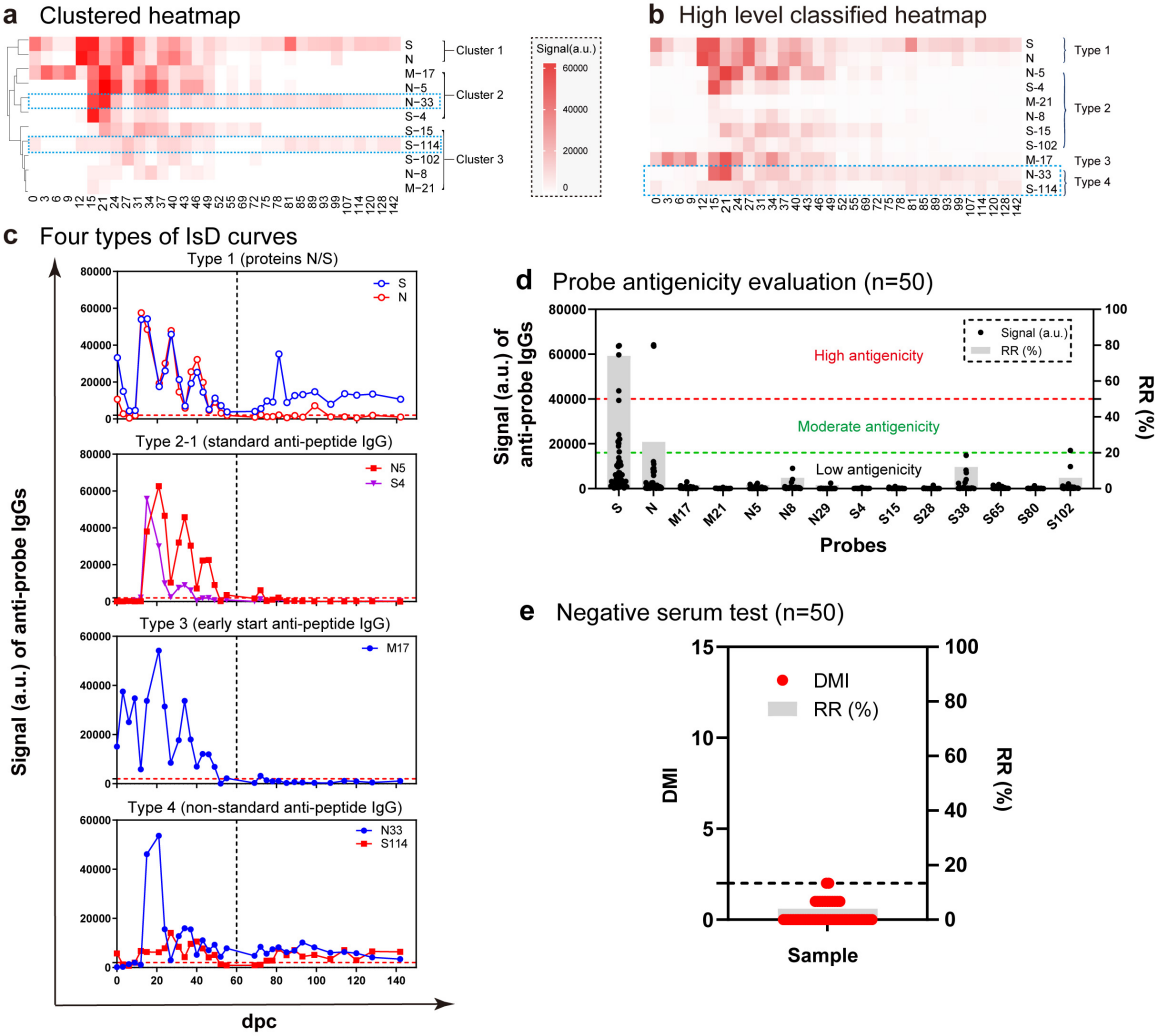
Optimization of PDCoV diagnostic epitopes. **(a)** Heatmap display of the “clustered” anti-probe IgGs for 11 SI probes of pig1. SI probes are divided into three clusters by clustering analysis: cluster 1 consists of 2 proteins; cluster 2 consists of 4 peptides; cluster 3 consists of 5 peptides. **(b)** Heatmap displaying of the “high-level analysis” of anti-probe IgGs for 11 SI probes of pig1. SI probes are divided into four types by high-level analysis: type 1 consists of 2 proteins from cluster 1; type 2 consists of 6 peptides (N5, S4, M21, N8, S15, and S102) from clusters 2 and 3; type 3 consists of M17 from cluster 2; type 4 consists of N33 and S114 from clusters 2 and 3, respectively. **(c)** Four types of IsD curve after “high level classification” of SI probe from pig1 are shown. Type2-1 only displays peptide of N5 and S4, while the IsD curves for the other peptides (M21, N8, S15, and S102) are shown in Supplementary Figure S11. **(d)** The antigenicity of candidate epitopes was tested using 50 PDCoV negative sera confirmed by virus neutralization tests (VNT). Candidate epitopes were divided into high (*RR* > 50%), moderate (20% < *RR* < 50%) and low (*RR* < 20%) antigenic epitopes according to their response rates (RR). **(e)** Diagnostic specificity test of PPHM_PDCoV_. Twelve epitopes with low antigenicity were used to test 50 PDCoV negative sera. When the digital microarray index (DMI) > 2, 2 samples tested positive, yielding a specificity of 96% [specificity = (number of total samples – number of negative samples)/number of total samples). The optimal cutoff value of DMI was set to 2, which yielded the highest diagnostic sensitivity and specificity (Supplementary Table S6). The red dots represent individual DMI values, and the gray bars indicate RR percentages, with a dashed line represents the DMI positivity threshold.

We first presented the response of all probes over time using a conventional heatmap (Supplementary Figure S5), where probes are arranged in the order of peptides' cleavage. Second, probes were divided into specific interaction (SI), NSI and Null based on IsD curves, and displayed in a classified heatmap (Figure 1d). Probes of the three types exhibit distinct characteristics: most SI probes begin to respond from 6 dpc and disappeared after 60 dpc. Some probes, such as N33 and S114, continued to respond after 60 dpc, while others, like M17, responded from 0 dpc. These two types were not observed in PCV2 (Figure 2c). The NSI probes show significant differences in their IsD curves, such as spike (*e.g*., S97) or continuous responding (*e.g*., E8; Supplementary Figure S6a). The signal of NSI probes are less than 2,000, and their IsD curves showed minimal change (Supplementary Figure S6b). As expected, we observed more diversity of SI probes in PDCoV compared to PCV2 (*e.g*., 5 SI probes) (Chen et al., 2022): 9 SI probes were identified for pig1.

Similarly, we obtained 20 and 15 SI probes through the longitudinal cohort sera of pig2 and pig3, respectively (Supplementary Figure S7). Analysis of the specific SI probes revealed that both pig2 and pig3 exhibited instances where adjacent peptides were simultaneously selected as candidate probes. For example, in pig2, four such peptide pairs were identified: M16/M17, N8/N9, S3/S4, and S101/S102. These peptides are sequentially adjacent, reflecting the design of the peptide library, where each peptide overlaps with its neighboring peptide by 10 amino acids (10-mer), as detailed in the Materials and Methods section. This overlapping strategy was intended to enhance antigenic coverage across the viral proteome. However, it also increases the likelihood that neighboring peptides may span the same or partially overlapping immunodominant epitopes, resulting in co-detection during serological screening. To reduce redundancy and improve the representativeness of selected epitopes for applications, we employed anti-peptide IgG sero-dynamics as a criterion to evaluate the immunogenicity of each peptide within these adjacent pairs. Specifically, we compared the relative intensity of anti-peptide IgG signals, the duration of the immune response, and the time of onset of antibody production. Based on these parameters, the peptide showing stronger immunogenicity and more distinct kinetic characteristics was retained as the representative candidate epitope for the corresponding region in both pig2 (Supplementary Figure S8) and pig3 (Supplementary Figure S9).

In total, 22 unique SI probes were identified as candidate diagnostic epitopes (Supplementary Table S2). Although the above results indicate that the IsDAED method is applicable to epitope identification for multi-protein pathogens, past experience suggests that only 5–12 epitopes are generally required for diagnostics. Accordingly, we further performed optimization screening of these 22 unique SI probes, as described below.

### Clustering and high-level analysis of SI probes facilitate the rapid identification of optimal diagnostic epitopes for multi-proteins pathogens

The IsDAED method is primarily used to identify SI peptides, but not all SI peptides are suitable as diagnostic probes. To select the best diagnostic probes from the 22 SI peptides, we first performed clustering analysis using the ComplexHeatmap from the R package (Gu, 2022). Taking pig1 as an example, the clustering heatmap results showed that 11 SI probes were divided into three clusters (Figure 2a). The IsD curves of the peptides in these clusters revealed distinct patterns. Proteins S and N belong to Cluster 1, with a response interval of 9 dpc to 60 dpc and signal values ranging from 0 to 60,000. M17, N5, N33, and S4 belong to Cluster 2, with a response interval of 12 dpc to 52 dpc and signal values ranging from 0 to 60,000. Cluster 3 includes five peptides: S15, S114, S102, N8, and M21, with a response interval of 12 dpc to 52 dpc and signal values ranging from 0 to 20,000 (Supplementary Figure S10).

Clustering heatmap is a methodological advancement over classified heatmap, enabling the rapid identification of probes with similar response intervals and signal values. However, clustering heatmap only relies on mathematics. As our understanding of the IsD curves of SI peptides deepens, current clustering analysis results are challenged: (1) The height and coverage area under the IsD curves of probes can reflect the “level of immunogenicity” (Xu et al., 2024). (2) When an immune response is triggered by external “antigens”, the IsD curves of probes will exhibit a “bell-shaped curve” over a period of time, which is called the “immune response interval.” The period of time after IgG levels return to their initial state is called the “non-immune response interval”. Based on clustering analysis results, it is not difficult to find that the immune response interval of pig1 is 0 dpc to 60 dpc, and the non-immune response interval is 60 dpc to 142 dpc.

Combing results from previous research, it is evident that anti-protein IgGs exhibit a sustained high response in both the immune and non-immune response intervals, while anti-peptide IgGs show a bell-shaped curve only during the immune response interval and are reactivated after subsequent stimulation (Chen et al., 2022; Xue et al., 2020). With reference to the epitope study experience of PCV2, we should prioritize candidate probes with immunogenicity and no response within the non-immune interval. However, clustering analysis cannot directly provide this type of grouping. For instance, N33 in Cluster 2 and S114 in Cluster 3 both maintain a low-level sustained response during the non-immune response interval, suggesting that they should be categorized together.

Therefore, we need to manually categorize SI probes of pig1 into four groups based on their “level of immunogenicity” and “response interval,” which we termed as high-level classification. High-level classification heatmap and IsD curves of probes respectively illustrate the characteristics of the following four types of probes (Figures 2b, c). Type 1 consists of the two proteins (N and S): the height and area under the IsD curves of N and S almost maintain consistent, reflecting their immunogenicity. Unlike the IsD curve of Cap (PCV2), a high-level sustained response in the immune response interval was not observed; instead, the IsD curves of N and S proteins are similar to anti-peptide IgG which exhibit a respond (bell-shaped curve) only during the immune response interval, and a low-level sustained response in the non-immune response interval. Additionally, anti-N IgG and anti-S IgG show a response at 0 dpc (signal values of 10,676 and 33,226, respectively), suggesting high antigenicity. Type 2 is the “standard” anti-peptide IgG group (including six peptides: M21/N5/N8/S4/S15/S102), which responds during the immune response interval and does not respond during the non-immune response interval, exhibiting complete IsD curves (Group2-1 only shows two representative peptides; Supplementary Figure S11). However, the height and area under the IsD curves of six peptides are inconformity, indicating differing levels of immunogenicity. At 0 dpc, all peptides did not respond, suggesting low antigenicity.

Type 3 is the early start anti-peptide IgG group (M17): the IsD curve is similar to Type 1, this peptide is characterized by a response at 0 dpc, possibly due to the presence of IgGs that non-specifically interact with this peptide in the pig. If M17 continues to show a high response in other serum samples, it can be considered to have high antigenicity. Type 4 is the “non-standard” anti-peptide IgG group (including two peptides: N33/S114): they exhibit a complete IsD curve during the immune response interval but a low-level sustained response (defined as continuous signal above the filter value of 2,000) in the non-immune response interval. The high-level classification method helps directly acquire optimal Type 1, 2 and 3 candidate epitopes of pig1, which feature immunogenicity and no response in the non-immune response interval, while Type 4 epitopes are excluded due to they response in the non-immune response interval.

Candidate epitopes of pig2 and pig3 were also determined according to the above analytical process (Supplementary Figures S12, S13). Combining screening results from three pigs, we preliminarily identified seven peptides from pig1, six peptides from pig2, and five peptides from pig3. All these peptides were categorized as Type 2 and 3, while the S and N proteins were classified into Type 1. All these probes were determined as candidate PDCoV epitopes through high-level classification. After excluding redundant peptides, a total of 12 peptides and 2 proteins were assembled to form the PPHM-#2 (Supplementary Table S3). Origin analysis of the 12 peptides revealed that M17 and M21 were recognized by all three pigs. S4 was shared by pig 1 and pig 3, and S102 was shared by pig 1 and pig 2. The remaining eight peptides (N5/8/29, S15/28/38/65/80) were identified in individual pigs. The differences in epitopes between the three pigs were due to the individual differences in the immune system upon stimulation (Mulè et al., 2024). As core components of the IsDAED method, clustering and high-level classification enable the efficient screening of immunogenic linear epitopes from a large number of candidate epitopes, which supports the rational selection of optimal diagnostic epitopes for multi-protein pathogens.

### Neutralizing antibody test assists in the exclusion of asymptomatic pig to achieve accurate elimination of highly antigenic probes

High antigenicity of probes is one of the primary causes of false positives in diagnosis. The clustering analysis identified candidate epitopes with immunogenicity but did not determine the level of antigenicity. PDCoV often leads to asymptomatic infection in pigs, so it is hard to collect real negative serum samples (Lu et al., 2023). Three reasons explain this situation. Blood has low viral loads, so viral nucleic acid cannot be steadily detected and brings negative amplification outcomes. The virus mainly stays in intestinal tissues and hardly circulates in peripheral blood, so serum contains little viral RNA. Long interval between sampling points also makes virus detection difficult.

VNT as one of the golden standards for diagnosis was used to select 50 PDCoV negative sera (*e.g*., log_2_ titer < 3) for antigenicity screening of 12 peptides and 2 proteins (PPHM-#2; Supplementary Figure S14a). They were categorized into candidate epitopes of high antigenicity (*R*R > 50%), moderate antigenicity (20% < *RR* < 50%), and low antigenicity (*RR* < 20%) based on the response rate (RR = number of positive samples/number of total samples; Figure 2d).

The screening results indicated that the RR of S protein is 74%, classifying it as a highly antigenic probe. The N protein had a RR of 26%, classifying it as a moderately antigenic probe. In addition, published reports have proven ELISA based on full-length N protein frequently cross-reacts with PEDV, which increases false positives (Ma et al., 2016). The RR of the other 12 peptides were all lower than 20%, classifying them as low antigenicity epitopes (Supplementary Table S4). In order to improve the diagnostic performance, 12 peptides with immunogenicity and low antigenicity were selected to form the diagnostic panel. We use digital microarray index (DMI) cutoff value of 2 to determine the positive or negative status of serum sample. The DMI value was defined as the sum of binary probe results, with 1 assigned to positive response and 0 to negative response. When setting DMI > 2 as the positive and DMI < 2 as the negative, only 2 out of 50 negative samples (*RR* = 4%) tested positive, corresponding to an RR of 4% (Figure 2e). This cut off standard is consistent with our previous studies (Chen et al., 2022; Pan et al., 2022). In the PDCoV study, DMI ≥ 2 yielded the highest diagnostic performance, with optimal balance between sensitivity of 88.9% and specificity of 100% (Supplementary Table S6). The 95% Clopper–Pearson confidence intervals (CIs) were 51.75%−99.72% for sensitivity and 29.24%−100% for specificity (Supplementary Figure S15).

After excluding highly and moderately antigenic S and N proteins based on neutralizing antibody-verified negative sera, a total of 12 non-redundant immunodominant peptides identified from *PDCoV_CZ2020* satrin were selected to constitute PPHM_PDCoV_ (Table 2). Sequence alignment indicated obvious variations only existed in S4 and S80. Overall, these 12 peptides shared more than 97.5% sequence identities with eight representative PDCoV strains, ensuring favorable application potential for broad-spectrum serological diagnosis (Supplementary Figure S16).

**Table 2:** PDCoV diagnostic epitopes determined by the challenge group of three piglets.

Pig ID	E^*^	M^*^	N^*^	S^*^	Number of epitopes
1	–	M17/21	N5/8	S4/15/102	7
2	–	M17/21	–	S38/65/80/102	6
3	–	M17/21	N29	S4/28	5
Total	–	M17/21	N5/8/29	S4/15/28/38/65/80/102	12

^*^E, M, N, and S represent the structural proteins of PDCoV. The table lists the specific epitopes and numbers of pig1/2/3 in each protein. A total of 12 non-redundant peptides were selected: pig1 recognized 7 peptides (M17, M21, N5, N8, S4, S15, S102); pig2 recognized 6 peptides (M17, M21, S38, S65, S80, S102); pig3 recognized 5 peptides (M17, M21, N29, S4, S28). Some epitopes (*e.g*., M17, M21) were recognized by all pigs, while others were unique or shared between two pigs. Overlapping peptides were merged for final analysis.

## DMI-nAb serology differentiates infection phases of field PDCoV

**Figure 3:**
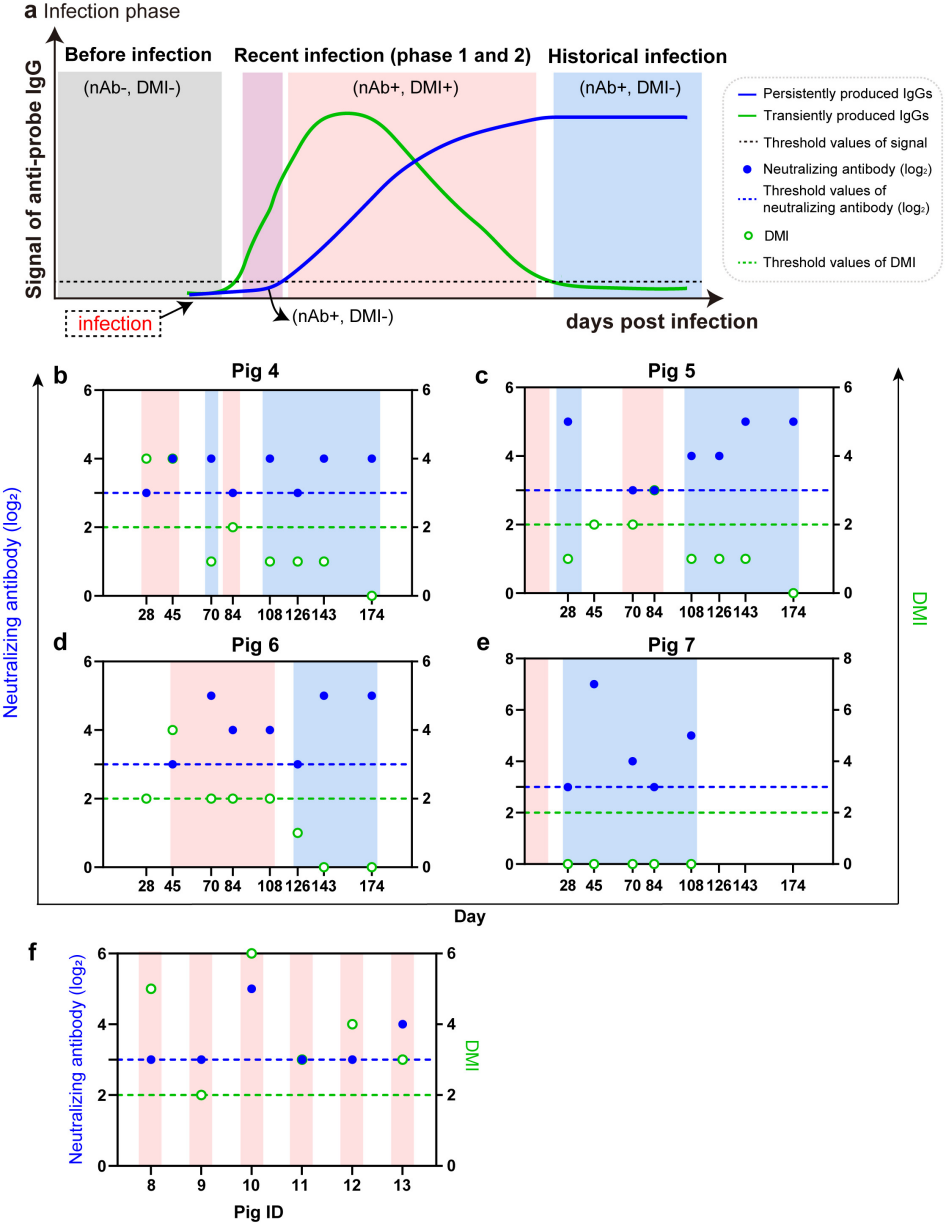
Temporal dynamics of IgGs responses across infection phases. **(a)** Schematic representation of anti-probe IgGs responses throughout four infection phases: before infection (gray), recent infection phase 1 (pink), recent infection phase 2 (red), and historical infection (blue). The right panel displays the figure legend. **(b–e)** Longitudinal changes in nAb (blue, left *y*-axis) and DMI (green, right *y*-axis) levels in the serum of individual pigs (pig 4/5/6/7), with shaded areas indicating different infection phases. Eight sera samples of 28–174 days of age from pig4/5/6 were detected respectively (the nAb results of pig5 missing 45 days of age and pig6 missing 28 days of age). Pig7 tested five serum samples from 28 to 108 days of age. **(f)** Distribution of viral load, nAb and DMI levels in the serum of pig 8/9/10/11/12/13 at a specific time point (175 days of age). Dotted lines represent threshold values for nAb and DMI positivity. nAb result of log_2_ ≥ 3 is positive, and log_2_ < 3 is negative. PPHM_PDCoV_ test result with DMI ≥ 2 is positive, and DMI < 2 is negative. All samples tested negative by PCR.

In the previous study of COVID-19, we confirmed that peptide-based IgGs could yield positive detection earlier than neutralizing antibodies (nAbs) after viral infection (Zheng et al., 2023). Furthermore, our previous work demonstrated the duration of IgGs responses detected by protein and peptide were significantly differences. The timing of their production and disappearance varies following viral infection (Xu et al., 2024). In the study, transiently produced IgGs are defined as IgGs produced during the early stage of the immune response. These IgGs typically persist for less than 60 days and are detectable using peptide antigens (*i.e*., anti-peptide IgGs). In contrast, persistently produced IgGs appear at the later stage of the immune response, persist for more than 200 days, and can only be detected using protein antigens (*i.e*., anti-protein IgGs). Given the high antigenicity of PDCoV S and N proteins in the present study, we used nAb levels to represent persistently produced IgG responses. The curves in Figure 3a illustrate the dynamic trends of transient and persistent IgG responses after virus infection, and the final infection status is determined by the combination of DMI and nAb results. Based on these two indicators, we further classified PDCoV infection into four sequential stages: before infection (nAb–, DMI–), recent infection phase 1 (nAb–, DMI+), recent infection phase 2 (nAb+, DMI+), and historical infection (nAb+, DMI–; Figure 3a). In real world infection cases, the duration of before infection and recent infection phase 1 are short, while recent infection phase 2 and historical infection are the most commonly observed.

Field samples exhibit complex PDCoV infection backgrounds, containing both symptomatic and asymptomatic cases (Lu et al., 2023). Without apparent clinical signs, asymptomatic infections pose a higher spillover risk compared with symptomatic infections. To investigate the natural infection dynamics of PDCoV in the field, sera were collected from four pigs (pig4/5/6/7) in the same column who had not been vaccinated with PDCoV from 28 to 174 days of age, with a total of eight sera per pig. Sampling was initiated at 28 days of age to eliminate interference from maternal-derived antibodies. Infection phase at each sampling point was classified according to the aforementioned infection staging model, and the corresponding classification results were annotated with distinct colors in the figures (Figures 3b–e).

At the first sampling (28 days of age), pig4 and pig6 were classified into recent infection phase 2, whereas pig5 and pig7 were assigned to the historical infection phase. These findings suggest that pig5 and pig7 acquired PDCoV infection prior to 28 days of age, and the restricted sampling frequency prevented us from capturing their recent infection phase. Longitudinal analysis revealed that pig4 and pig5 experienced reinfection during the monitoring period, whereas pig6 and pig7 underwent only a single infection episode. Importantly, all four pigs showed no clinical symptoms throughout the experiment, and all blood samples were PCR-negative. Collectively, these results demonstrate that all four pigs underwent asymptomatic PDCoV infection.

To further validate the feasibility and accuracy of our DMI-nAb combined staging system under field conditions, six additional 175-day-old unvaccinated pigs (pig8–13) were randomly collected from the field. According to their DMI and nAb results, all six animals were classified into recent infection phase 2 (Figure 3f). Consistent with the previous cohort, none of these pigs displayed observable clinical symptoms, yet all tested negative by qRT-PCR despite positive serology. As asymptomatic infection is defined as laboratory-verified pathogen infection via serology or molecular testing without clinical manifestations, negative RT-PCR cannot rule out prior infection owing to transient viral shedding and sampling-caused false-negative results (Boyton and Altmann, 2021; Sajal et al., 2024). Given our PPHM_PDCoV_ targets epitopes verified via pig challenge experiments, specific seroconversion reliably confirms authentic PDCoV exposure. Collectively, these six pigs were confirmed as true asymptomatic PDCoV infections, highlighting the superiority of our peptide-based assay over conventional qRT-PCR for discovering asymptomatic infections.

## Both inactivated and S-based subunit vaccines of PDCoV only induced weak TPIs responses

**Figure 4:**
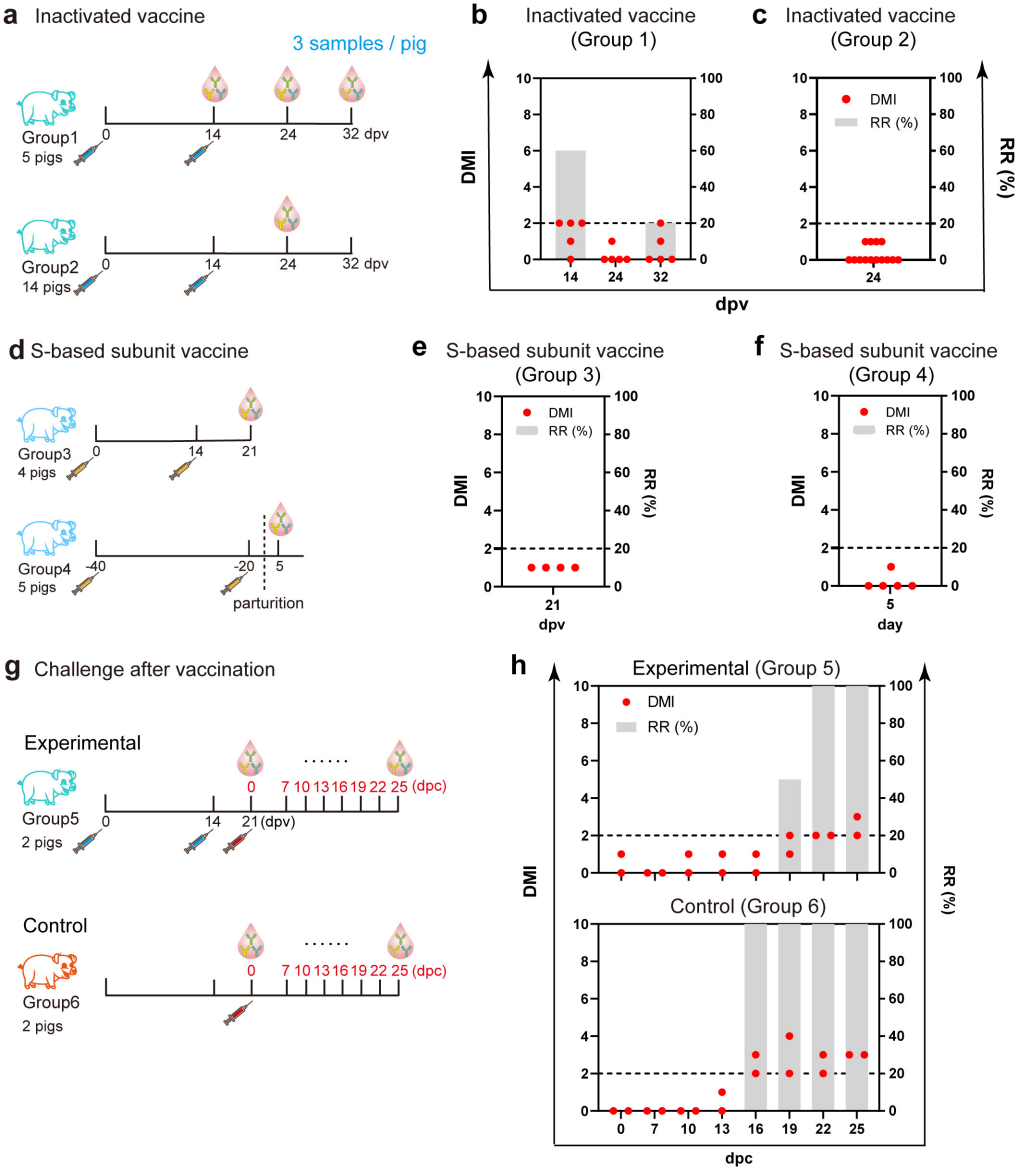
The weak response of anti-peptide IgGs after PDCoV vaccines immunization. **(a)** Experimental design for the inactivated vaccine group: pigs at 5 days of age were divided into two groups (Group 1 with five pigs, Group 2 with 14 pigs) and immunized intramuscularly the same dose of PDCoV inactivated vaccine (dose: 1 ml/10^7.5^ TCID_50_/ml per piglet) at 0 and 14 days (*i.e.*, 0 days post- vaccination, dpv). Group 1 collected sera samples at 14 dpv, 24 dpv, and 32 dpv, while Group 2 collected sera samples only at 24 dpv. The results of PPHM_PDCoV_ for the inactivated vaccine immunization serum samples Group 1 (*n* = 5) **(b)** and Group 2 (*n* = 14) **(c)**. **(d)** Experimental design for the S-based subunit vaccine group: four 5-day-old piglets were immunized intramuscularly the same dose of S subunit vaccine (2 ml × 200 μg per piglet) at 0 and 14 days, with serum collected at 21 dpv (Group 3). Group 4 consisted of five suckling pigs from sows vaccinated with S-based protein vaccine at 40 and 20 days prior to parturition, and serum was collected from the suckling pig at 5 days of age. The results of PPHM_PDCoV_ for the S-based subunit vaccine immunization serum samples Group 4 (*n* = 4) **(e)** and Group 5 (*n* = 5) **(f)**. **(g)** Experimental design for the challenge after vaccination group: pigs at 5 days of age were divided into two groups (Group 5 with two pigs, Group 6 with two pigs). Group5 (experimental) was vaccinated following the Group 1 protocol and challenged at 21 dpv (dose: 10 ml × 10^6.0^ TCID_50_/ml per piglet), while Group 6 (control) challenged directly. Sera were collected at 0, 7, 10, 13, 16, 19, 22, and 25 days (*i.e.*, days post- challenge, dpc). **(h)** The results of PPHM_PDCoV_ for experimental Groups 5 and control Group 6 showing that Group 5 tested positive at 19 dpc, while Group 6 tested positive earlier, at 16 dpc. The red dots represent individual DMI values, and the gray bars indicate RR percentages, with a dashed line represents the DMI positivity threshold.

Our previous work has documented the development of PDCoV inactivated vaccines and S-based subunit vaccines (Li et al., 2023). Herein, we comparatively analyzed serological IgGs dynamic profiles induced by PDCoV vaccination and viral challenge. We first conducted immunization experiments using the inactivated vaccine (Zheng et al., 2023). Group 1 consisted of five pigs that are 5 days of age, which were vaccinated with the inactivated vaccine at 0 dpv and 14 dpv (*i.e.*, 0 days post-vaccination, 0 dpv), and sera samples were collected at 14 dpv, 24 dpv and 32 dpv, with a total of 3 sera per piglet (Figure 4a). The data showed that no nAbs were produced at 14 dpv, while the titers of nAbs were ranged from 1: 32 to 1: 128 at 24 dpv and 32 dpv, indicating successful vaccination and the ability to produce high-titer nAbs (Supplementary Figure S14b). Further, the PPHM_PDCoV_ results indicated that only three pigs tested positive at 14 dpv (*RR* = 60%). All five pigs were negative at 24 dpv (*RR* = 0). Only one pig tested positive at 32 dpv (*RR* = 20%; Figure 4b, Supplementary Figure S17a). In theory, 24 dpv and 32 dpv are the peak of antibody response, and a large number of anti-peptide IgGs should be observable. However, the response of anti-peptide IgGs immunization by PDCoV inactivated vaccine was low, leading us to speculate that it might be an individual difference with a small number of samples.

To eliminate the impact of individual difference on detection results, in Group 2 we expanded the number of samples from 5 to 14 pigs using the same immunization protocol as Group 1 but sera samples were only collected at 24 dpv (Figure 4a). The titers of nAbs in the 14 pigs ranged from 1: 64 to 1: 256 indicating successful vaccination. The PPHM_PDCoV_ test results showed that all 14 pigs were negative (*RR* = 0), indicating that PDCoV inactivated vaccine does not induce TPIs at 24 dpv (Figure 4c, Supplementary Figures S14c, S17b). This result is consistent with those observed in Group 1. In Group 1, the pigs were in the IgG immune response phase at 14 dpv, with a RR of 60% detected by PPHM_PDCoV_. However, three pigs showed a low signal of anti-peptide IgG (< 10,000). At 24 dpv, the IgG level may have undergone a decline. The RR is 20% at 32 dpv, only one pig has immune response, it may have been due to infection, which subsequently activated immune memory. Notably, significant individual immune differences were observed among the pigs. Therefore, we conclude that PDCoV inactivated vaccine immunization only induces a weak anti-peptide IgGs response at 14 dpv, and anti-peptide IgGs disappeared 24 dpv.

We also collected samples from pigs immunized with the S-based subunit vaccine (Groups 3 and 4). Group 3 was immunized with the S-based subunit vaccine at 0 dpv and 14 dpv, with samples collected at 21 dpv (Figure 4d). PPHM_PDCoV_ results showed all pigs were negative (*RR* = 0; Figure 4e). However, the titers of nAb ranged from 1: 64 to 1: 256 (Supplementary Figure S14d). Group 4 consisted of suckling pigs from sows vaccinated with S-based protein vaccine at 40 and 20 days before parturition, respectively. Serum was collected from the suckling pig at 5 days of age (Figure 4d). PPHM_PDCoV_ results showed all pigs were negative (*RR* = 0; Figure 4f). However, the titers of nAb ranged between 1: 32 to 1: 64 (Supplementary Figure S14e). The nAb test results indicated that the S-based subunit vaccine was successfully immunized. Analysis of the anti-peptide IgG response revealed that S102 responded in Group 3 and S38 responded in Group 4, but these responses were deemed negative due to *DMI* < 2 (Supplementary Figures S17c, d). This suggests that the S-based subunit vaccine also induces only a weak anti-peptide IgG response.

Based on these findings, we conclude that both the PDCoV inactivated and the S-based subunit vaccine are capable of inducing high titers of nAbs, yet the response of anti-peptide IgGs are weak and rapidly diminished. This suggests that both vaccines primarily stimulate the production of IgGs against conformational epitopes, while the IgGs against linear epitopes (*i.e*., anti-peptide IgGs) were barely induced. According to our previous research on PCV and *Peste des petits ruminants virus* (PPRV), this is advantageous for DIVA (Chen et al., 2022; Xue et al., 2020). Then, we designed a challenge after vaccination experiment with the PDCoV inactivated vaccine. In Group 5, two pigs were selected as experimental group and immunized following the same protocol as Group 1, and they were challenged at 21 dpv. Sera samples were collected at 0, 7, 10, 13, 16, 19, 22, and 25 dpc, with a total of 8 sera samples per pig. Group 6 served as control group, which was challenge without vaccine (Figure 4g). The PPHM_PDCoV_ test results showed that Group 5 was positive at 19 dpc (Figure 4h), indicating viral infection. In contrast, Group 6 tested positive at 16 dpc (Figure 4h), confirming the success of the challenge experiment. These differences reflect the partial protective effect of the inactivated vaccine against PDCoV infection. Regarding the anti-peptide IgG response, signal values in Group 5 were significantly lower than those in Group 6 (Supplementary Figure S18). In summary, conventional PDCoV vaccines trigger minimal anti-peptide IgG production, while wild-type PDCoV infection induces strong peptide-specific IgGs. The PPHM_PDCoV_ assay can potentially serve as a DIVA tool to distinguish vaccinated animals from naturally infected ones. Further studies with expanded sample sizes and more vaccine formulations are required for full validation and field adaptation.

## TPIs indicate that PDCoV causes asymptomatic infections in pigs

**Figure 5:**
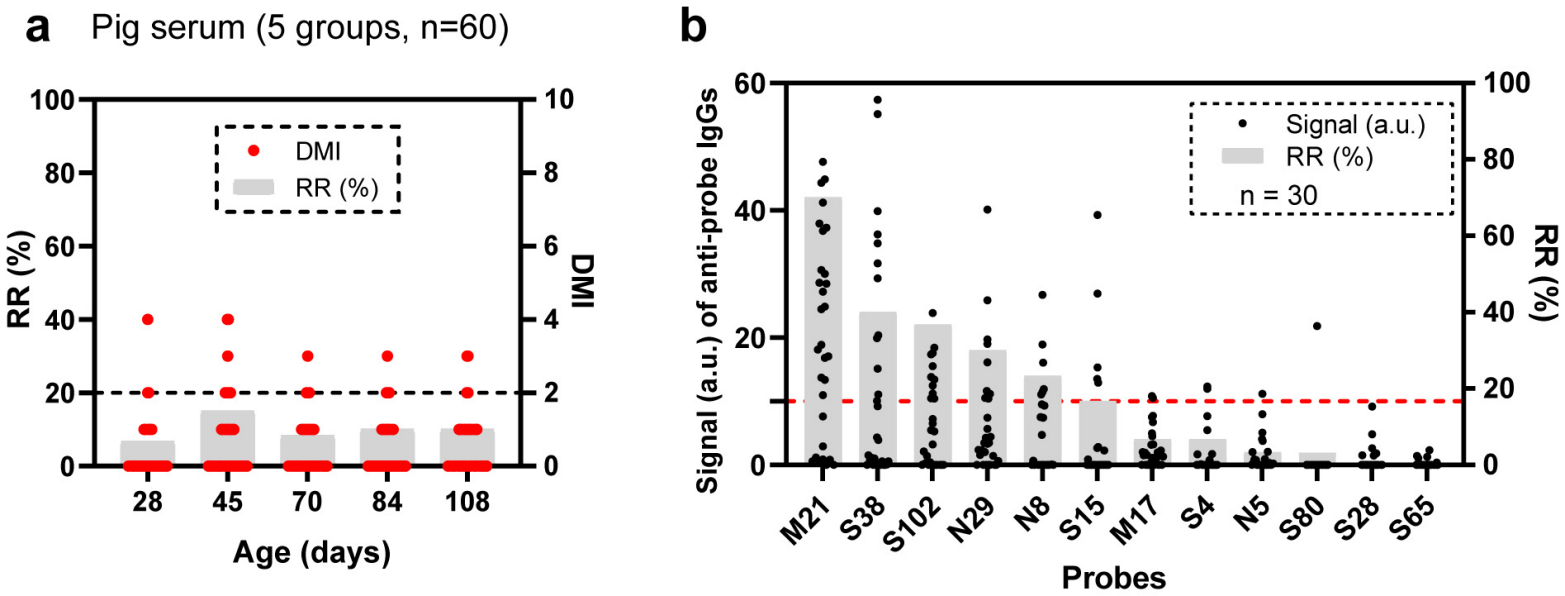
Detection of PDCoV prevalence between pig samples in the field. **(a)** A total of 60 pigs were tested using the PPHM_PDCoV_ at 28, 45, 70, 84 and 108 days of age respectively. The RR increased with the age of the pigs and remained stable after 70 days of age. The red dots represent individual DMI values, and the gray bars indicate RR percentages, with a dashed line represents the DMI positivity threshold. **(b)** Signal of 12 anti-probe IgGs. Black dots represent individual signal, and gray bars indicate RR for each probe. The red dashed line represents the signal positivity threshold.

PDCoV has been widely spread in the world since the first discovery, especially in southern China (Zhang et al., 2024). To investigate the current prevalence of PDCoV, we selected 60 unvaccinated pigs and collected serum samples at 28, 45, 70, 84, and 108 days of age. PPHM_PDCoV_ detection showed the infection rates at the above time points were 6.7%, 15%, 8.3%, 10% and 10%, respectively. Seropositive rates with 95% Wald confidence interval are listed (Table 3). The rate peaked at 15.00% (95% *CI*: 5.98 %−24.02%) on day 45 vs. 10.00% (95% CI: 2.42%−17.58%) on day 84. Fisher's exact test indicated no significant difference between the two time points (*P* = 0.599). Notably, all pigs remained clinically asymptomatic across the observation period (Figure 5a). In total, 300 serum samples were obtained from the 60 pigs across five time points, and 30 samples yielded positive results via PPHM_PDCoV_. These positive sample results included repeated positive measurements from the same individuals at different time points; after recalculation at the individual pig level, 15 out of 60 pigs were DMI-positive at any sampling point, with an overall animal-level prevalence of 25% (15/60). Considerable differences were found in the response levels of diverse anti-peptide IgGs among positive samples (Figure 5b). Given that all pigs remained unvaccinated and clinically healthy, these cases were classified as asymptomatic PDCoV infections. Considering the potential zoonotic spillover risk of PDCoV, we further screened 311 human sera samples from two types of human serum samples across three different regions to check for possible spillover (Supplementary Table S5). The results of PPHM_PDCoV_ showed negative (*RR* = 0; Supplementary Figure S19). Taken together, these field surveillance data demonstrate prevalent asymptomatic PDCoV infection in field pig samples, while no evidence of PDCoV zoonotic spillover to human populations was detected in the sampled cohorts.

**Table 3:** Seropositive rates and 95% confidence intervals at different sampling time points.

Time point (days)	Positive/total samples	Seropositive rate (%)	95% Wald confidence interval
28	4/60	6.67	0.43%−12.91%
45	9/60	15.00	5.98%−24.02%
70	5/60	8.33	1.43%−15.23%
84	6/60	10.00	2.42%−17.58%
108	6/60	10.00	2.42%−17.58%

Fisher's exact test showed no significant difference in seropositive rates between day 45 and day 84 (*P* = 0.599).

## Discussion

The study used PDCoV as an example to evaluate the applicability of the IsDAED method for accurately identifying epitopes in multi-protein pathogens. Overall, we optimized our experimental strategy from two main aspects: sample selection and methodological improvement of the IsDAED approach. For sample selection, we established a 3-day interval longitudinal serum cohort from challenged pigs rather than vaccinated animals, as viral challenge induces broader and stronger anti-peptide IgG responses (Chen et al., 2022). Frequent sampling also better captured more nuanced trends in IsD curves. Three pigs were chosen for both methodological and ethical reasons: the 30% response rate threshold required at least one responder among three individuals, enabling initial screening of 22 candidates and final selection of 12 optimal epitopes for PPHM_PDCoV_ (Table 2), with results validated in two additional pigs (Figure 4h). Consistent with our previous work, three animals are adequate for initial diagnostic epitope screening, and this design also minimized animal usage in compliance with welfare guidelines. Statistical analysis revealed that three pigs yield an 87.5% detection rate for epitopes present in 50% of infected animals, which meets the 80% screening threshold. In addition to optimizing the serum cohort, we also improved the quality control strategy for negative serum samples. We further applied VNT to strictly screen negative sera and exclude asymptomatic PDCoV carriers. Unlike SARS-CoV-2 studies that define negative sera merely by health background (Xu et al., 2023), PDCoV asymptomatic infection limits this simple approach. Instead of routine PCR screening used in COVID-19 (Kent et al., 2022), we relied on persistent nAb to determine infection status using a single sample, improving accuracy and reducing sampling workload.

Following the optimization of sample selection, we further upgraded the workflow of the IsDAED method. For the improvement of IsDAED, we added clustering analysis and high-level analysis. Clustering analysis can group probes with similar response trends and signal characteristics. On this basis, high-level analysis further considers probe immunogenicity and immune response intervals. This analytical process greatly shortens the time for screening and selecting optimal epitopes from a large number of candidate probes, and helps define standard IgG response patterns for rapid diagnostic epitope identification. We also added a procedure to remove highly antigenic probes. The original IsDAED method can evaluate probe immunogenicity according to IsD curves (Xu et al., 2024), but it cannot rule out probes with high antigenicity. The improved workflow effectively increases the reliability of epitope screening, and the optimized IsDAED method is suitable for epitope screening of many other multi-protein pathogens.

Although the improved IsDAED method facilitates epitope determination, linear epitopes still have inherent limitations in serological detection. Linear epitopes are commonly used in immunological assays for antibody recognition. However, as they consist of continuous amino acid residues, they are more prone to NSI and non-reproducible interactions (NRI) in serological detection (Pan et al., 2022). To avoid false positives in diagnostics, the exclusion of highly antigenic probes is crucial. Given the complexity of individual immune backgrounds, we believe that testing probes with a serum library unassociated with immune events (*i.e.*, negative serum) can reveal the antigenicity of the probes (Xu et al., 2023). In the reaction system, as long as the diversity of IgGs and probes are sufficiently large, unpredictable NSI and NRI will inevitably occur (Pan et al., 2022; Xu et al., 2023). Testing against negative serum libraries can calculate an RR for the probes, which can help judge the level of probe antigenicity. The RR for the S and N proteins was 74% and 26% respectively, classifying them as high and moderate antigenic epitopes. These probes also showed a low-level sustained response in the non-immune response interval, which may reflect the poor quality of the expressed S and N proteins. To more accurately detect IgGs against conformational epitopes, we therefore employed the VNT, which relies on the recognition of wild-type viral structures and is less affected by protein expression.

In our previous studies on *Peste des Petits Ruminants Virus* based on the PPHM platform, both peptides and proteins were used to detect IgGs in longitudinal serum samples (Xue et al., 2020; Xu et al., 2024). We observed significant differences in the duration of IgGs responses detected by proteins and peptides. Anti-protein IgGs typically have a longer duration (usually more than 200 days), whereas anti-peptide IgGs have a shorter duration (usually less than 60 days). Based on the differing response durations, we define TPIs as follows: IgGs that are produced during the early stage of the immune response. These IgGs typically persist for less than 60 days and are detectable using peptide antigens (*i.e*., anti-peptide IgGs). In contrast, persistently produced IgGs appear at the later stage of the immune response, persist for more than 200 days, and can only be detected using protein antigens (*i.e*., anti-protein IgGs). It is important to note that not all anti-peptide IgGs are TPIs. However, in our PDCoV peptide epitope combination, all anti-peptide IgGs disappeared within approximately 60 days, which aligns with the definition of TPIs. One limitation of this work is that we did not continue serum collection after 142 dpc, so anti-protein IgGs persistence exceeding 200 days could not be validated via our PDCoV animal data.

Based on the above definition of TPI and persistently produced IgGs, we further analyzed why PDCoV vaccines fail to induce obvious anti-peptide TPI responses. Several immunological mechanisms can explain this phenomenon. First, inactivated and subunit vaccines often preserve native protein conformation, preferentially activating B cells that recognize conformational or discontinuous epitopes, whereas linear epitopes (represented by peptides) depend on processing and presentation via MHC class II pathways and may receive limited T-cell help in the vaccine context. Second, vaccine formulations and adjuvants may not optimally promote Th1/Th2 responses required for sustained anti-peptide IgG production. Third, natural infection provides prolonged antigen exposure and a broader array of T-cell epitopes, facilitating stronger and more diversified B-cell responses against linear epitopes. Importantly, this discordance between vaccine-induced and infection-induced anti-peptide IgG profiles offers a clear serological signature for differentiating infected from vaccinated animals (DIVA), enhancing the practical utility of the PPHM platform.

In our 2022 study on PCV2, we observed that certain vaccine formulations led to the lack of anti-peptide IgG production, whereas wild-type virus infections consistently triggered these antibodies (Chen et al., 2022). Similarly, in the case of PDCoV, wild-type infections consistently induced anti-peptide IgG responses, whereas the PDCoV vaccine tested in the study failed to generate these responses. These inherent immunological differences between vaccination and natural infection lead to distinct TPI response patterns, which can be exploited to establish a novel DIVA strategy for PDCoV. Previous research has discovered that some vaccines do not induce anti-peptide IgGs (*i.e.*, IgG of linear epitope), but only produce anti-protein IgGs (*i.e.*, IgG of conformational epitope) (Chen et al., 2022). A similar phenomenon was observed in the study, where PDCoV inactivated and S-based subunit vaccines only induced a weak response of anti-peptide IgGs, yet the titers of nAb were high (Figure 4, Supplementary Figures S14b–e). This indicates that these vaccines primarily induce IgGs of conformational epitopes. Based on the anti-peptide IgG responses observed in the challenge after vaccination groups (Figure 4h, Supplementary Figure S17), we believe that natural infection will generate a more active anti-peptide IgG response, similar to that seen in the challenge group. DIVA can be determined by detecting the presence or absence of anti-peptide IgGs using PPHM_PDCoV_. Previous studies on PCV2 and PPRV used both anti-peptide IgGs and anti-protein IgGs for DIVA (Chen et al., 2022; Xue et al., 2020). In the study, the IsD curves of anti-S and anti-N proteins show an unusual pattern and exhibit a bell-shaped response during the immune response interval, and a low-level sustained response in the non-immune response interval. Therefore, DIVA for PDCoV only focuses on the response of anti-peptide IgGs. Although only two piglets were used in the post-vaccination challenge group, serial longitudinal sampling offsets the small sample size. This study mainly proposes a new DIVA concept, and comprehensive verification will be conducted in follow-up research.

Beyond DIVA application, combined detection based on PPHM_PDCoV_ and nAb also enables precise discrimination of different infection phases and identification of asymptomatic infection. It is known that VNT is the golden standard for detecting nAb. Co-positive DMI and nAb indicate recent infection, whereas negative DMI with positive nAb suggests historical infection (Figures 3a–e). Moreover, we established a persistent asymptomatic infection model of PDCoV based on the following evidence. First, maternal antibodies in pigs typically wane by 3–4 weeks of age, whereas individuals such as pig5 and pig6 showed increasing DMI levels at both 28 and 45 days of age, suggesting an infection-induced immune response rather than residual maternal antibodies (Shen et al., 2024). Second, the peptide epitopes used in the PPHM_PDCoV_ platform were all derived from validated experimental infections and are capable of sensitively detecting infection-induced IgG responses. Finally, the sampled pigs were from farms without recent PDCoV outbreaks or vaccination history, and showed no clinical symptoms at the time of sampling, supporting the presence of persistent asymptomatic infections rather than passive immunity or latent infection. Compared with traditional detection methods, transiently produced IgGs (TPIs) can identify early asymptomatic PDCoV infections effectively, and avoid false-negative results caused by low viral loads.

Given the potential risk of PDCoV cross-species spillover, we further evaluated the feasibility of PPHM_PDCoV_ for human serological surveillance and analyzed the limitations of the study. Although the spillover of PDCoV in Haitian children is very limited (Lednicky et al., 2021), a large-scale interspecific transmission may occur in a country with over 700 million annual pig output. Assuming shared diagnostic epitopes between humans and pigs, our PPHM_PDCoV_ could be employed to monitor the prevalence of PDCoV in human populations, serving as an effective early warning surveillance method. The limitations of this study include: (1) Confirmed PDCoV-positive human serum samples were unavailable, preventing direct validation of the platform in human samples. (2) No human PDCoV epitope studies have been conducted; the PPHM_PDCoV_ uses pig-derived epitopes, which may not fully capture human PDCoV antibody responses, making it unclear whether the platform can detect cross-species infections. (3) Although no PDCoV-specific IgGs were detected in samples we tested, low-level or epitope-shifted infections cannot be excluded. Furthermore, the human cohort size (*n* = 311) was not based on a formal sample-size calculation for a predefined prevalence threshold, which may limit the ability to definitively rule out very low-level zoonotic transmission. Future serosurveys should incorporate power-based sampling designs and high-risk human populations (*e.g*., breeders, veterinarians, and live-market employees) to strengthen epidemiological conclusions.

As a typical model pathogen with prevalent asymptomatic infection, PDCoV provides valuable insights for similar infectious diseases. The PPHM platform established in the study is also suitable for other infectious diseases with asymptomatic infections, such as *brucellosis* (Tian et al., 2024), *Human Papillomavirus* (Soheili et al., 2021) and *Epstein-Barr Virus* (Smatti et al., 2018). Future work should expand sample collection in PDCoV-endemic regions and high-risk populations with known exposure to pigs. This will help assess the platform's sensitivity and field applicability. In addition, identifying PDCoV-specific epitopes in human infections will help reveal host-specific immune responses and satisfy cross-species transmission monitoring. These efforts will enhance the diagnostic performance of the PPHM_PDCoV_ platform and support its broader application in asymptomatic infection research.

## Conclusions

The study established a PPHM_PDCoV_ for the serological detection of asymptomatic PDCoV infections through TPIs. By refining the IsDAED method with clustering and high-level analytical strategies, diagnostic epitopes were efficiently identified across multi-proteins pathogens. The platform revealed a 15% asymptomatic infection rate in pigs at 45 days of age. These findings indicate that PPHM_PDCoV_ provides a reliable and scalable approach for early infection detection, DIVA, and zoonotic risk surveillance in PDCoV control.

## Data Availability

The original contributions presented in the study are included in the article/Supplementary material, further inquiries can be directed to the corresponding authors.
