# Supplementary figures and images for: Detection of ongoing asymptomatic Porcine deltacoronavirus infections via transiently produced IgGs using protein-peptide hybrid microarray
Source: Front Microbiol. 2026 Jun 24;17:1846662. doi: 10.3389/fmicb.2026.1846662 (PMC13341950; doi:10.3389/fmicb.2026.1846662)

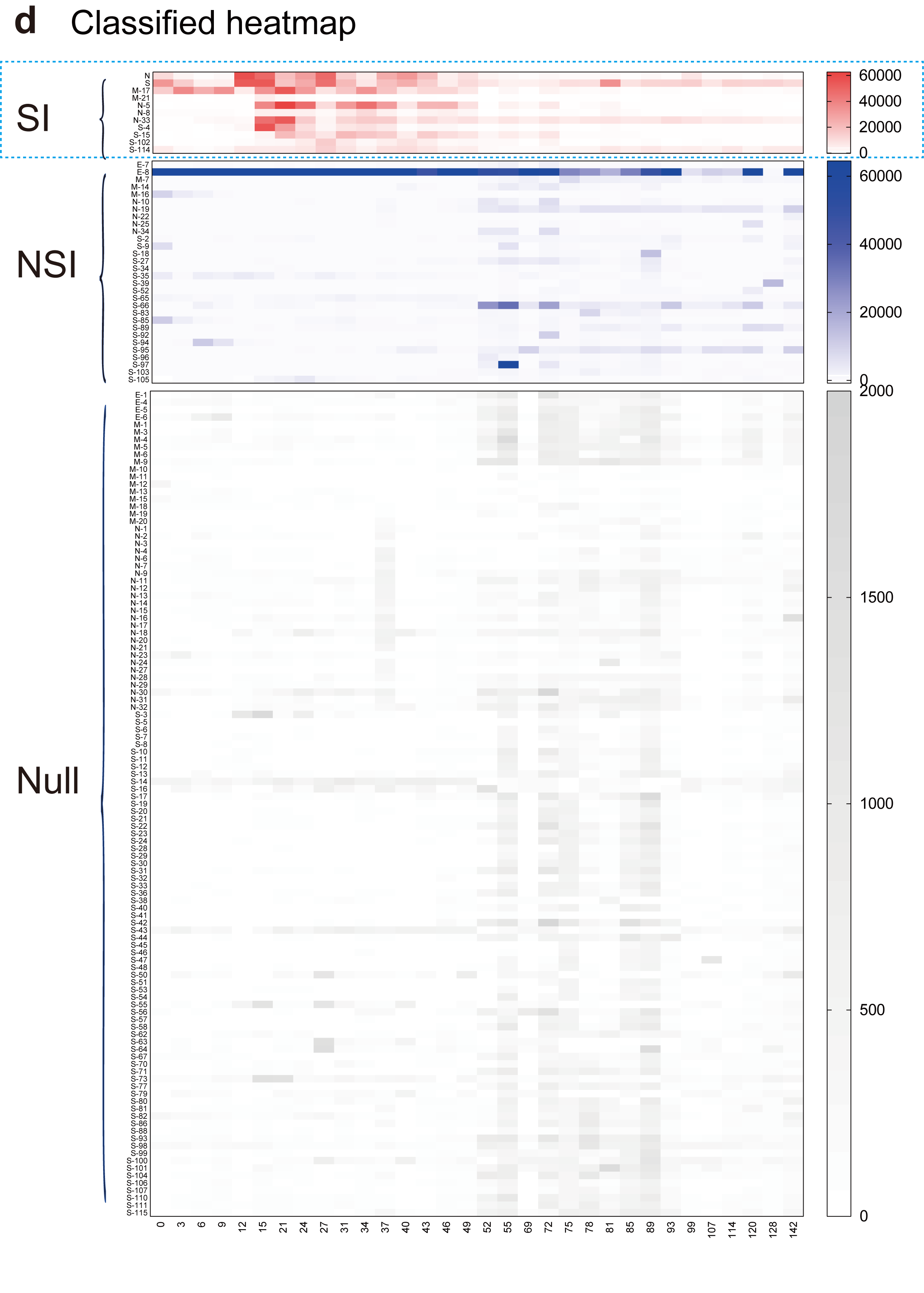

Supplement: Supplementary file 1 [file Image_1.TIF]
