# Supplementary material for: Detection of ongoing asymptomatic Porcine deltacoronavirus infections via transiently produced IgGs using protein-peptide hybrid microarray
Source: Front Microbiol. 2026 Jun 24;17:1846662. doi: 10.3389/fmicb.2026.1846662 (PMC13341950; doi:10.3389/fmicb.2026.1846662)
Supplement: Supplementary file 2 [file Table_1.DOCX]

**Detection of ongoing asymptomatic *Porcine Deltacoronavirus* infections via transiently produced IgGs using protein-peptide hybrid microarray**

Mengyu Li^1,2‡^, Jizong Li^3‡^, Lan Yang^2^, Zhiwei Li^4^, Dayong Gu^5^, Xiaoting Xu^6^, Yezi Liu^4^, Xiaoyu Li^1,2^, Wei Wang^3^, Bin Li^3*^, Hongwei Ma^1,2*^

^1^School of Nano-Tech and Nano-Bionics, University of Science and Technology of China, Hefei 230026, China

^2^Division of Nanobiomedicine, Suzhou Institute of Nano-Tech and Nano-Bionics, Chinese Academy of Sciences, Suzhou 215123, China

^3^Institute of Veterinary Medicine, Jiangsu Academy of Agricultural Sciences, Key Laboratory of Veterinary Biological Engineering and Technology Ministry of Agriculture, Jiangsu Key Laboratory for Food Quality and Safety-State Key Laboratory Cultivation Base of Ministry of Science and Technology, Nanjing, China

^4^Clinical Laboratory Center, People's Hospital of Xinjiang Uygur Autonomous Region, Urumqi, 830001 Xinjiang, China

^5^Department of Laboratory Medicine, Shenzhen Institute of Translational Medicine, The First Affiliated Hospital of Shenzhen University, Shenzhen Second People's Hospital, Medical Innovation Technology Transformation Center of Shenzhen Second People's Hospital, Shenzhen University, Shenzhen 518035, China

^6^Department of Radiation Oncology, The First Affiliated Hospital of Soochow University, Suzhou 215000, China

‡ These authors contributed equally to this work.

^*^Correspondence to: H. Ma; E-mail: [hwma2008@sinano.ac.cn](mailto:hwma2008@sinano.ac.cn) and Bin. Li; E-mail: libinana@126.com


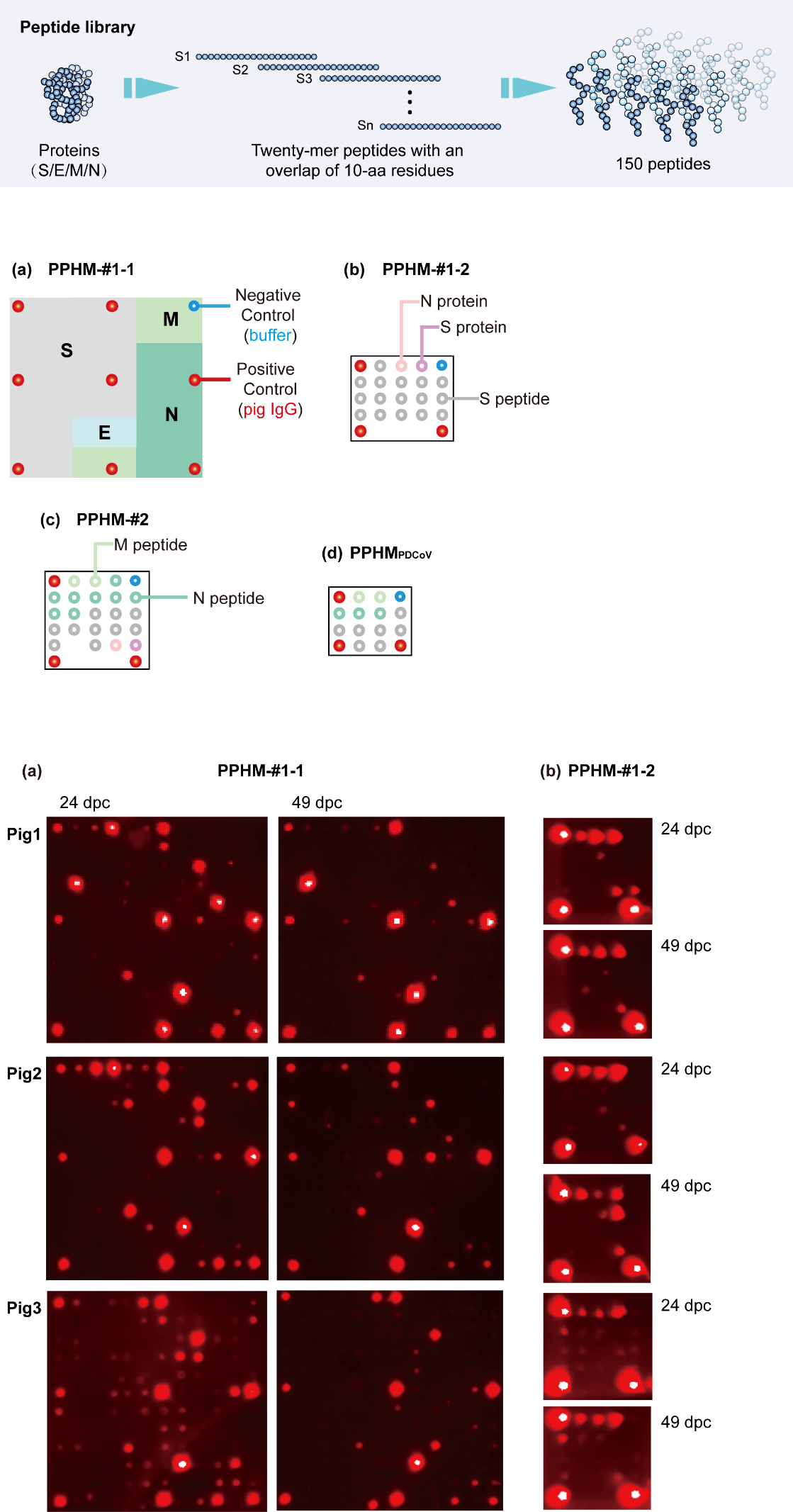


**Figure S1. Construction of the overlapping peptide library.** The S, E, M, and N proteins of PDCoV were divided into 20-mer peptides with 10-amino-acid overlaps, yielding 150 peptides for linear epitope identification.


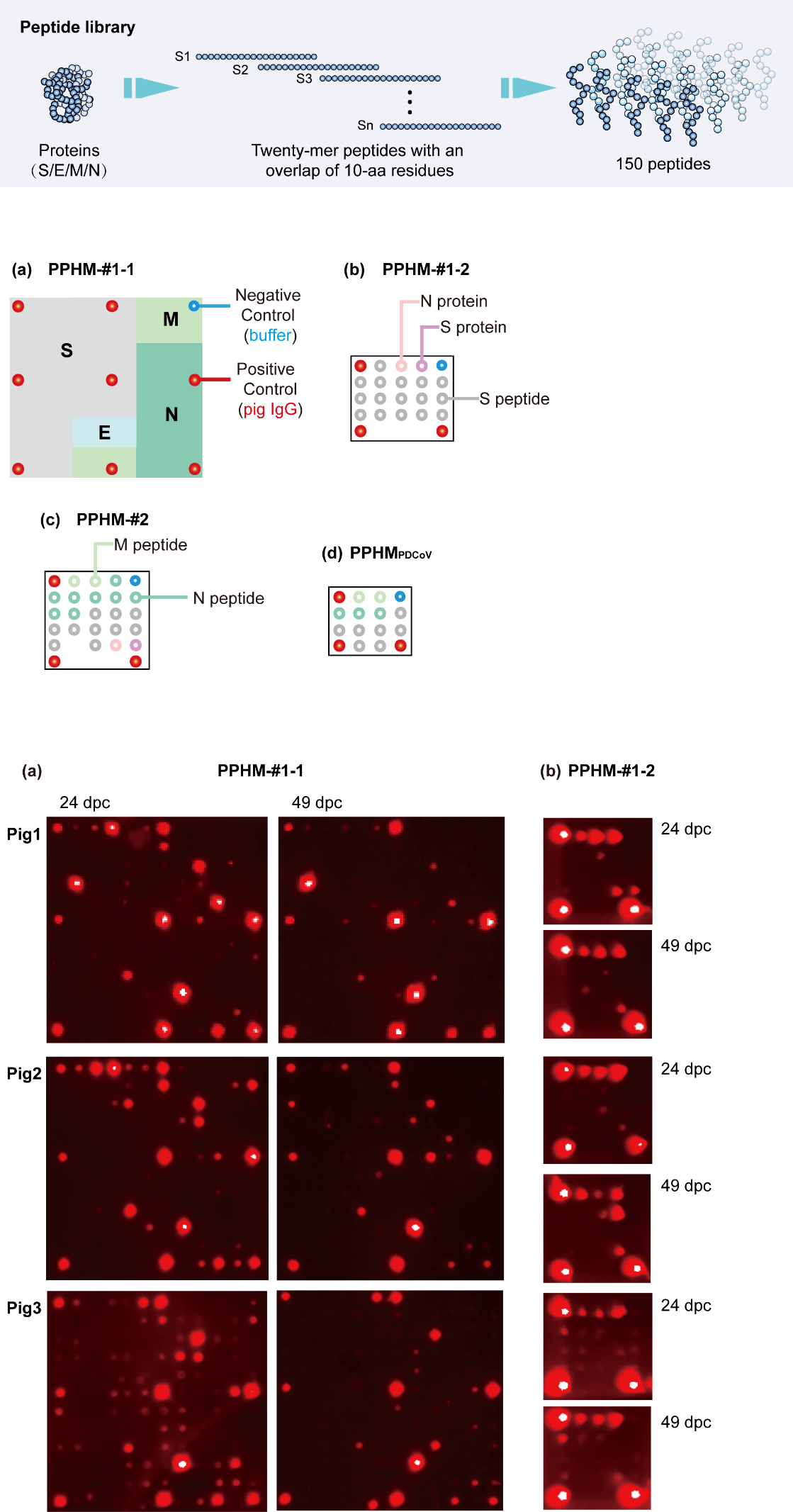


**Figure S2. Schematic diagram of the PDCoV protein-peptide hybrid microarray (PPHM) design.** (a) Layout of the macroarray PPHM-#1-1, containing 134 detection peptides (6 derived from the E protein, 19 from the M protein, 33 from the N protein, and 76 from the S protein) along with positive (pig IgG) and negative (buffer) controls. (b) Layout of the microarrays PPHM-#1-2, containing the remaining 16 peptides from the S protein, full-length recombinant N and S proteins, and the same positive and negative controls. (c) Layout of the microarrays PPHM-#2, incorporating full-length S and N proteins, 20 selected diagnostic peptides (M17/21, N5/8/9/10/19/29/30/33, S4/15/28/38/65/80/86/89/102/114) , and the controls.. (d) Final integrated PPHM_PDCoV_ microarray layout, containing only the most specific probes: 12 selected peptides (M17/21, N5/8/29, S4/15/28/38/65/80/102), pig IgG as positive controls, and the printing buffer as negative controls.


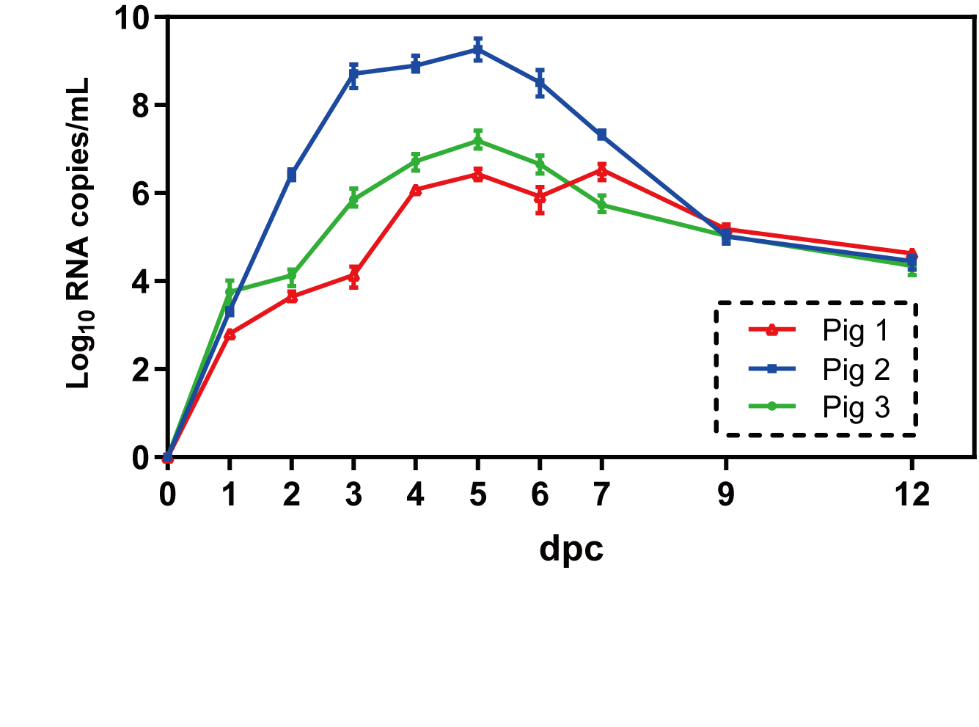


**Figure S3. Fluorescence quantitative PCR analysis of PDCoV fecal viral shedding in three pigs from the challenge group**.


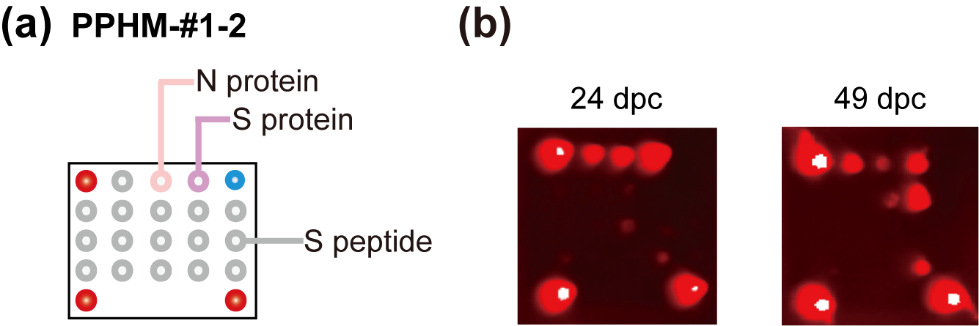


**Figure S4. Composition of PPHM-#1-2 and screening results of pig1 at 24 dpc and 49 dpc.** (a) Schematic layout of the PPHM-#1-2. The array contains N and S protein, 16 peptides were all from S protein. Positive control are marked in red and negative control are marked in blue at the corners of the array. (b) Representative results of anti-probe IgG responses from pig 1 at 24 dpc and 49 dpc. The red fluorescent signals indicate binding of anti-probe IgG to specific epitopes on the array.

**Figure S5. Conventional heatmap of pig1’s anti-probe IgGs.** All probes are arranged sequentially from top to bottom according to proteins and peptides, the proteins are N and S respectively, and peptides ordered based on the cleavage sequence of E, M, N, and S proteins.


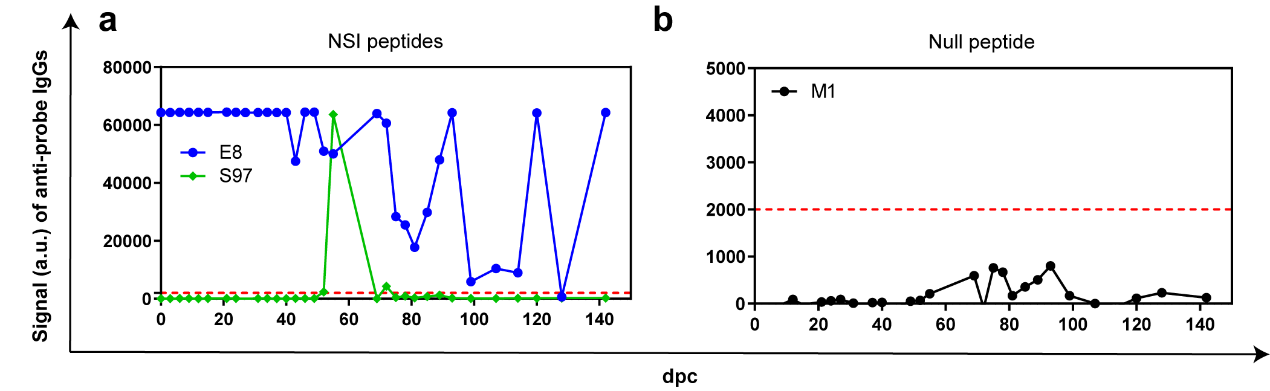


**Figure S6. Representation of NSI and Null peptides for pig1.** (a) Two typical representatives of NSI peptides. E8 is an NSI peptide associated with sustained response, while S97 is an NSI peptide of the spike type. (b) A typical representative of Null peptides. M1exhibits a signal below the detection limit (Signal < 2000) and a relatively flat IsD curve. The red dashed line represents the signal positivity threshold.


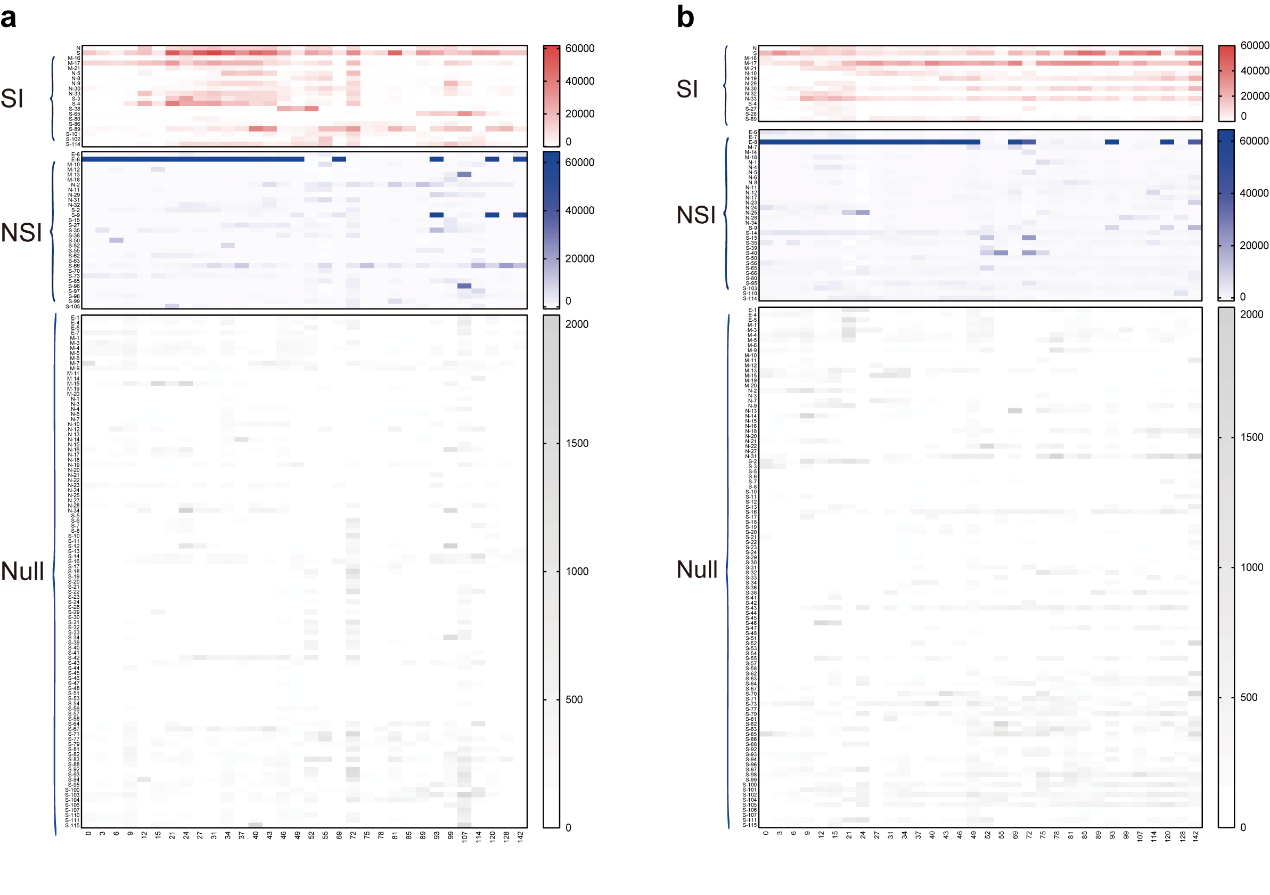


**Figure S7. Classified heatmaps of pig2 (a) and pig3’s (b) anti-probe IgGs (2 proteins + 150 peptides).** For pig2, there are 20 SI probes (13.2%), 31 NSI probes (20.4%), and 101 Null probes (66.4%). For pig3, there are 15 SI probes (9.9%), 34 NSI probes (22.4%), and 103 Null probes (67.8%).


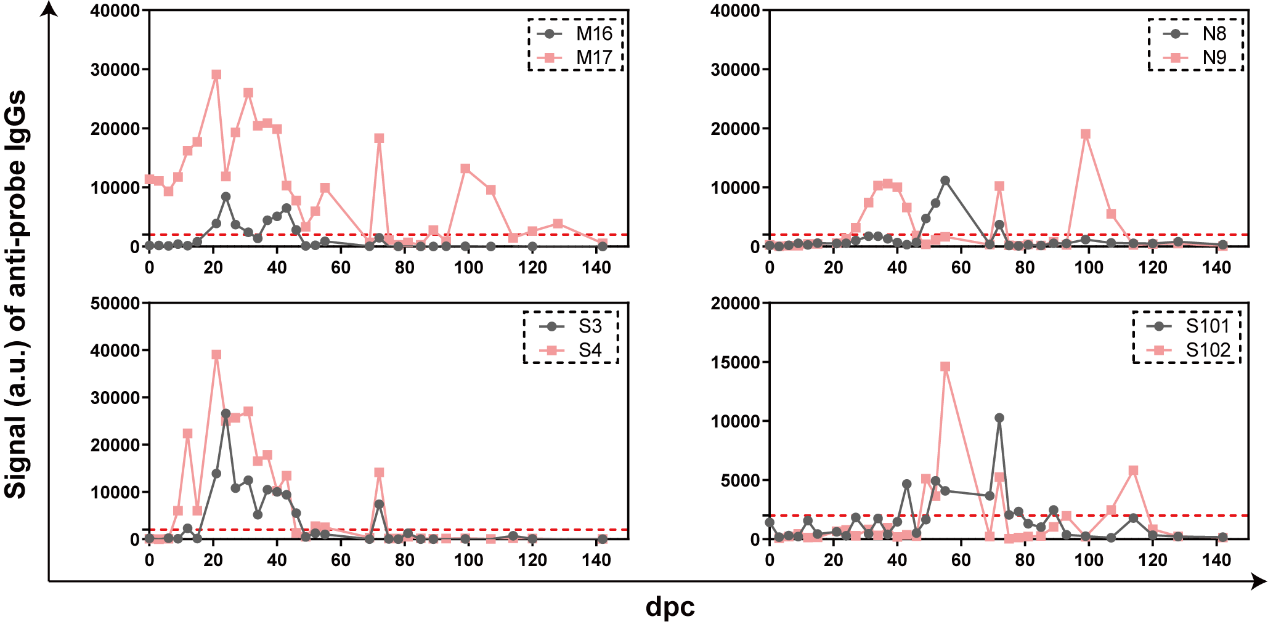


**Figure S8.** **Representative** **IgG sero-dynamics of** **adjacent peptides for pig2.** Each of the four selected peptide pairs (M16/M17, N8/N9, S3/S4, and S101/S102) from pig2 features a 10 amino acid overlap due to the design of the peptide library. For each pairs, the peptide which detect higher IgG signal, exhibited a broader response duration, or more distinct IgG kinetic features was considered more immunogenic and was selected as the representative epitope in the region (the pink curve in the figure). The red dashed line represents the signal positivity threshold.


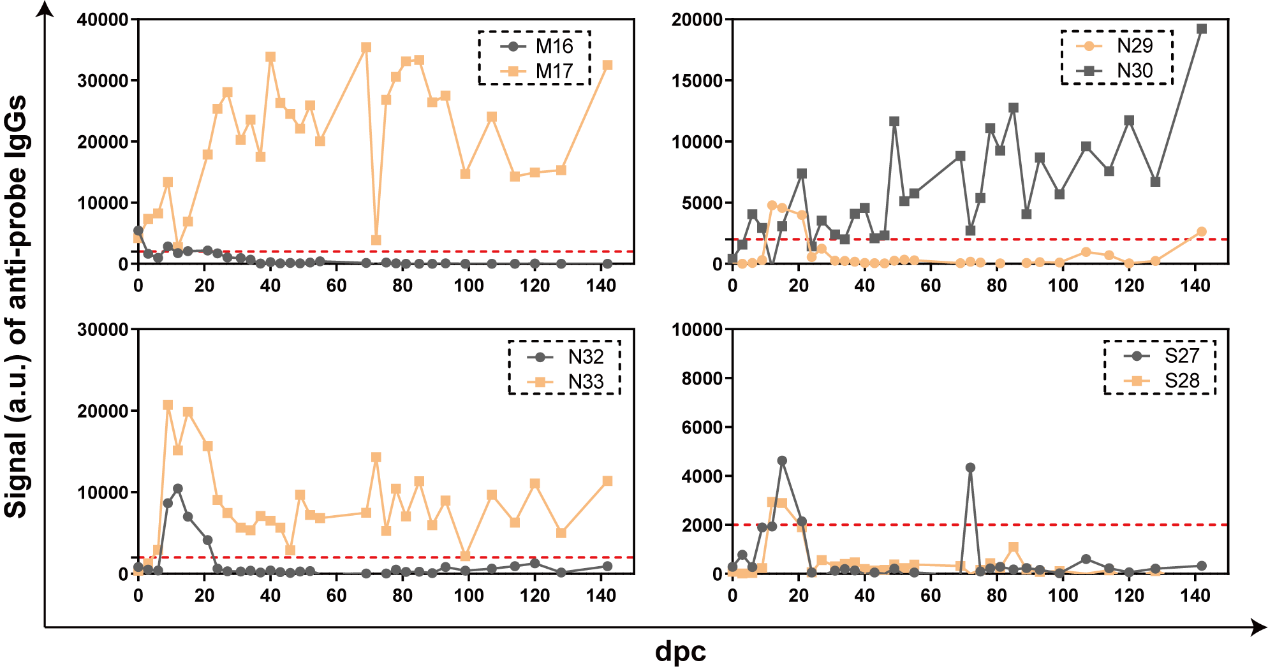


**Figure S9.** **Representative** **IgG sero-dynamics of** **adjacent peptides for pig3.** Each of the four selected peptide pairs (M16/M17, N29/N30, N32/N33, and S27/S28) from pig3 features a 10 amino acid overlap due to the design of the peptide library. For each pairs, the peptide which detect higher IgG signal, exhibited a broader response duration, or more distinct IgG kinetic features was considered more immunogenic and was selected as the representative epitope in the region (the yellow curve in the figure). The red dashed line represents the signal positivity threshold.


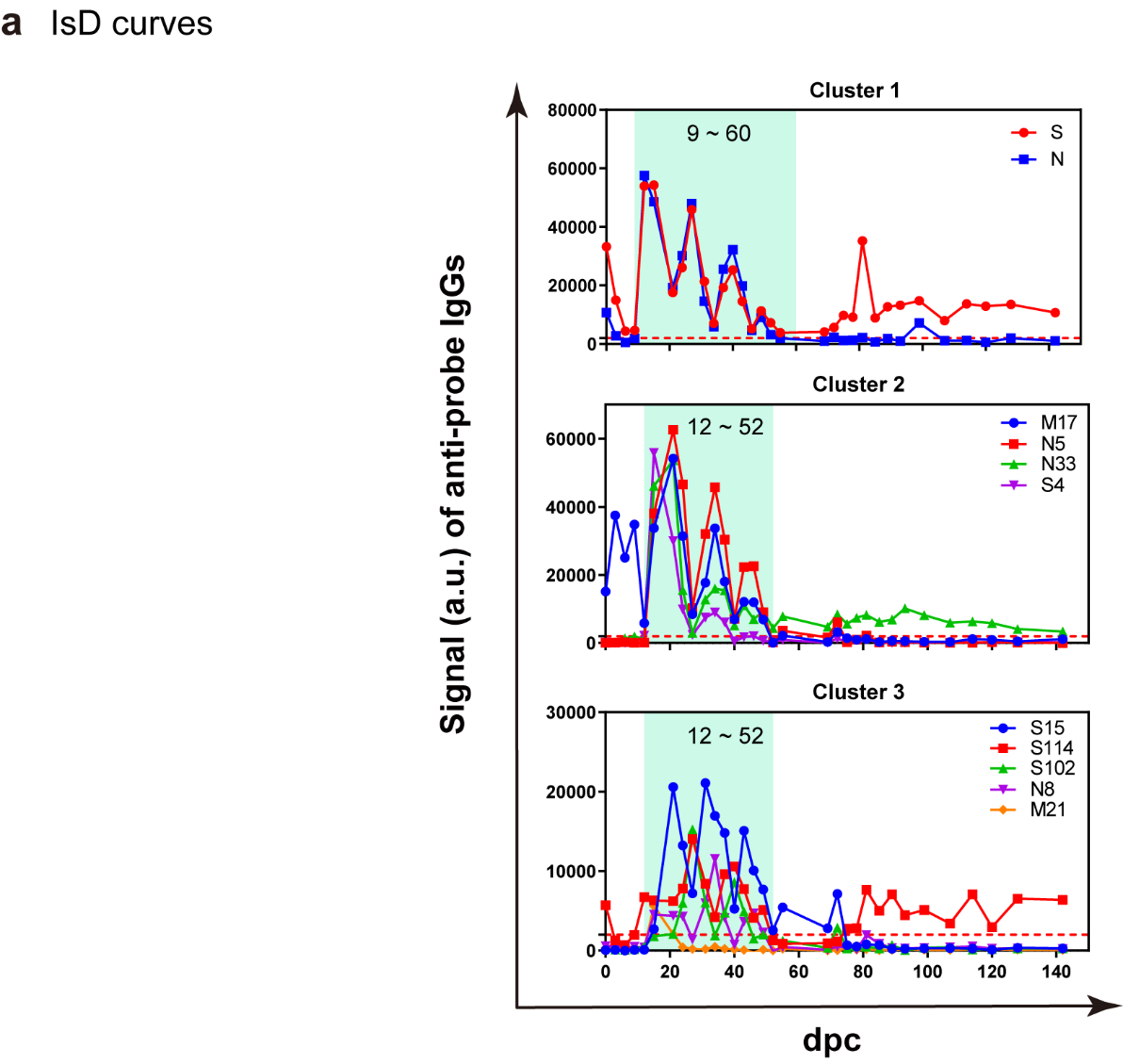


**Figure S10. Presentation of IsD curves for the “clustering heatmap” of anti-probe IgGs corresponding to the SI probes of pig1.** Cluster 1 includes two proteins (S and N), while Clusters 2 and 3 comprised of 4 and 5 peptides, respectively. The shaded regions highlight immune response intervals (9–60 dpc for Cluster 1 and 12–52 dpc for Clusters 2 and 3) where antibody show significant responses. The red dashed line represents the signal positivity threshold.


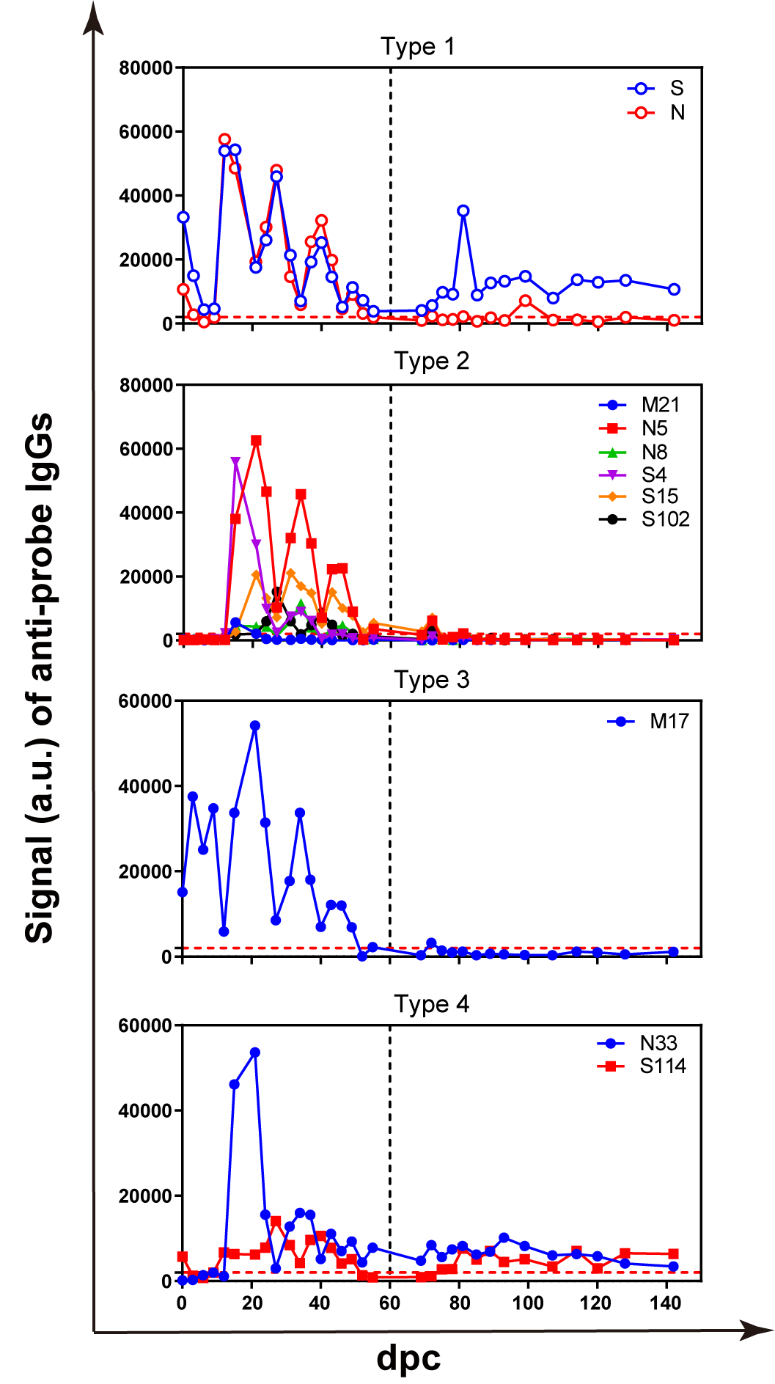


**Figure S11. Presentation of IsD curves for the “high-level classification” of anti-probe IgGs corresponding to the SI probes of pig1.** Type 1 includes two proteins (S and N), type 2 includes six peptides, type 3 only contains one peptide (M17), and type4 includes two peptides. The red dashed line represents the signal positivity threshold.


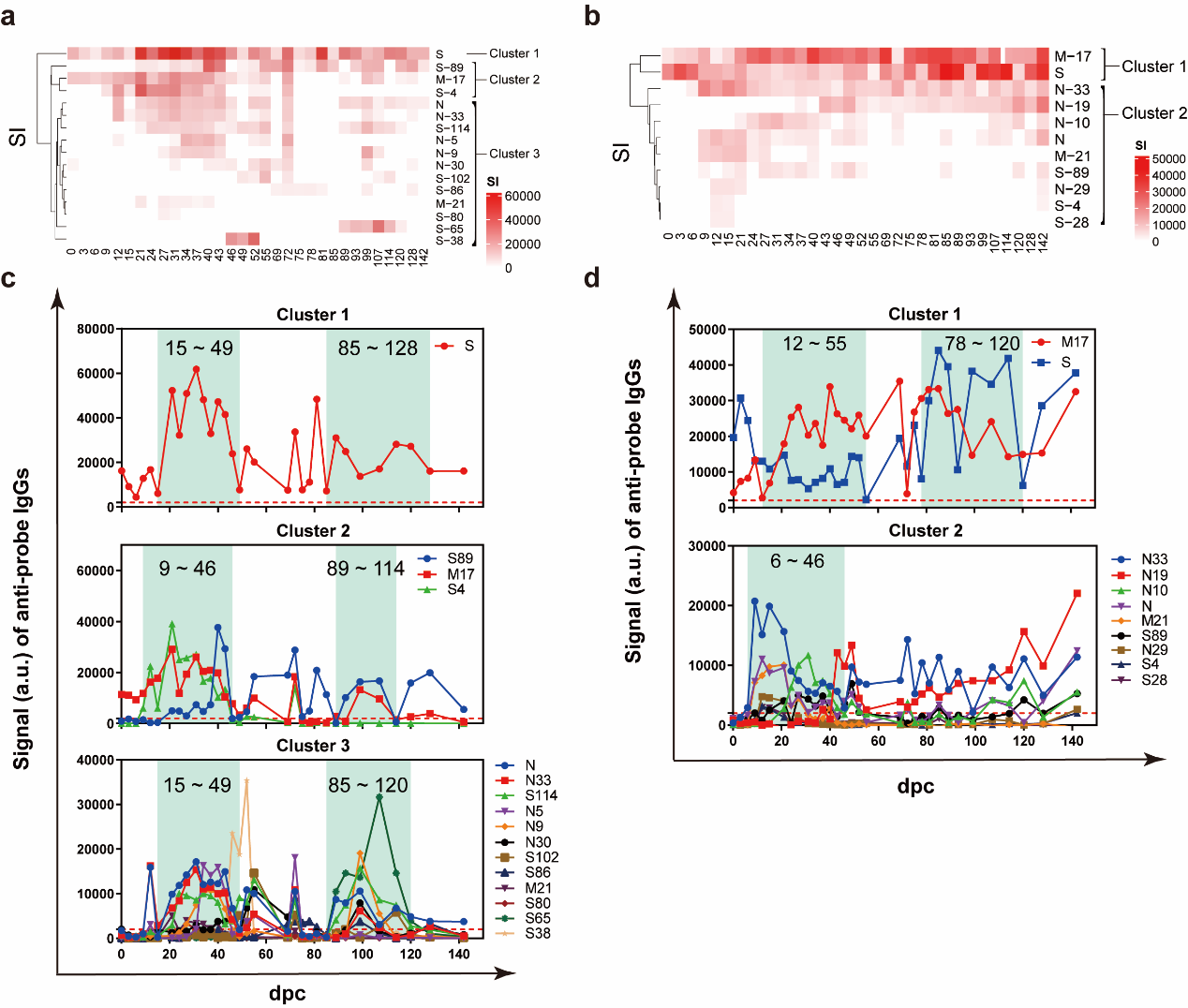


**Figure S12.** **Overview of the “cluster analysis” of anti-probe IgGs responses in pig2 and pig3.** Heatmap display of the "clustered" anti-probe IgGs for 16 and 11 SI probes of pig2 (a) and pig3 (b) respectively. SI probes of pig2 are divided into three clusters by clustering analysis: Cluster 1 consists of one proteins; Cluster 2 consists of three peptides; Cluster 3 consists of one protein and eleven peptides. SI probes of pig3 are divided into two clusters by clustering analysis: Cluster 1 consists of one protein and one peptide; Cluster 3 consists of one protein and eight peptides. Presentation of IsD curves for the “clustering heatmap” of anti-probe IgGs corresponding to the SI probes of pig2 (c) and pig3 (d). The shaded regions highlight immune response intervals of pig2 (15–49 dpc for Cluster 1 and 3 and 9–46 dpc for Clusters 2) , while pig2 also respond in non-immune response intervals (85–128 dpc for Cluster 1, 89–114 dpc for Clusters 2, 85–120 dpc for Clusters 2). The immune response intervals of pig3 is 12–55 dpc for Cluster 1 and 6–46 dpc for Clusters 2, non-immune response intervals is 78~120 dpc for Cluster 1. The red dashed line represents the signal positivity threshold.


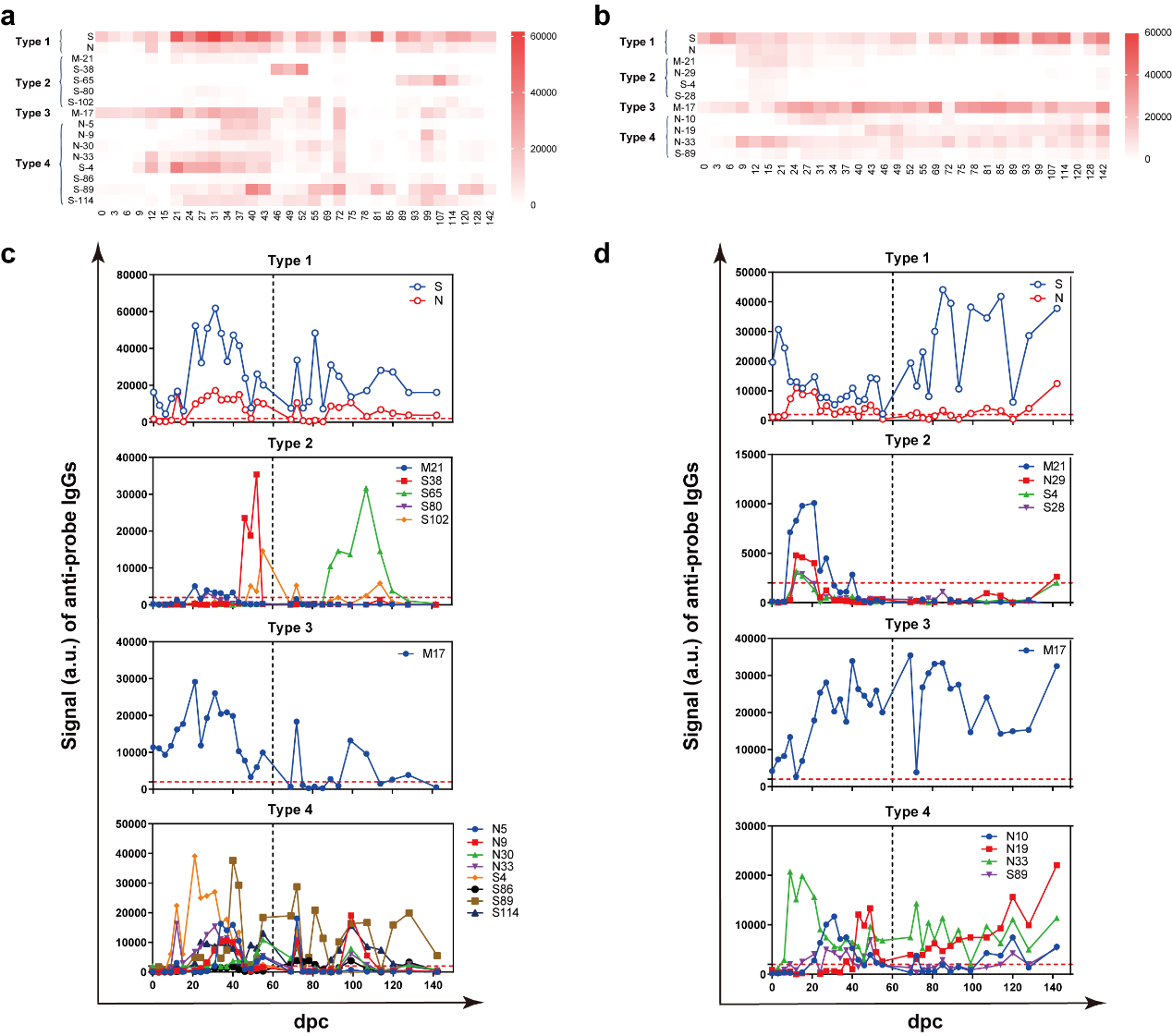


**Figure S13.** **Overview of the “high-level analysis” of anti-probe IgGs responses in pig2 and pig3.** Heatmap displaying of the "high-level analysis" of anti-probe IgGs for 16 SI probes of pig2 (a) and 11 SI probes of pig3 (b). Pig2 are divided into four types by high-level analysis: Type 1 consists of two proteins from Cluster1 and Cluster3, respectively; Type 2 consists of five peptides (M21, S38, S65, S80 and S102) from Cluster3; Type 3 consists of M17 from Cluster2; Type 4 consists of eight peptides (N5, N9, N30, N33, S4, S86, S89 and S114) from Cluster2 and Cluster3, respectively. Pig3 are also divided into four types by high-level analysis: Type 1 consists of two proteins from Cluster1 and Cluster2, respectively; Type 2 consists of four peptides (M21, N29, S4 and S28) from Cluster2; Type 3 consists of M17 from Cluster1; Type 4 consists of four peptides (N10, N19, N33 and S89) from Cluster2. Presentation of IsD curves for the “high-level classification” of anti-probe IgGs corresponding to the SI probes of pig2 (c) and pig3 (d). The red dashed line represents the signal positivity threshold.


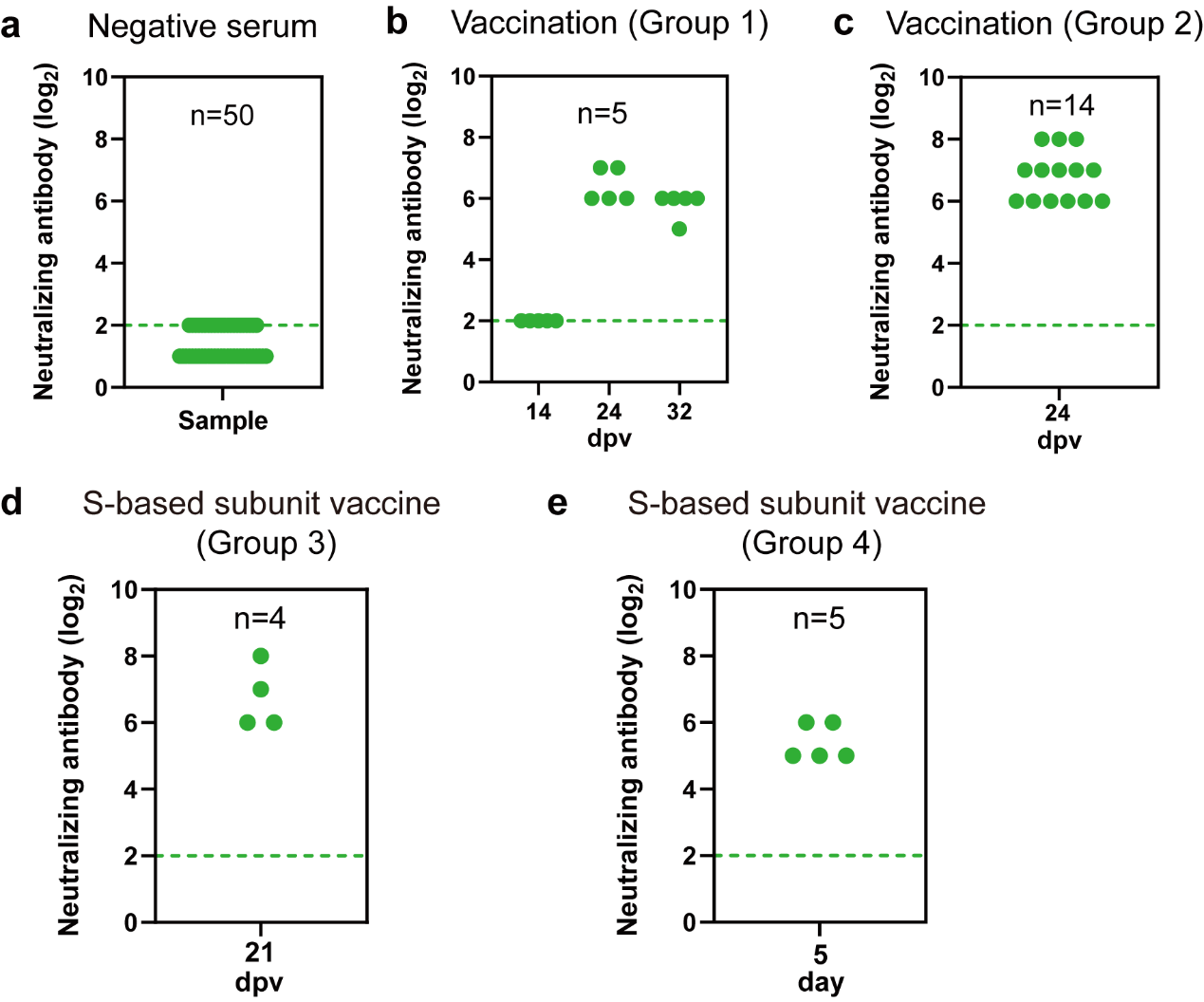


**Figure S14. The detection of neutralizing antibody titers in PDCoV from diverse samples.** (a) 50 serum samples tested negative for neutralizing antibodies in PDCoV. (b) Group 1 (PDCoV inactivated vaccine immunized samples): five pigs tested negative for neutralizing antibodies in PDCoV at 14 days post-vaccination (dpv), and positive at 24 dpv and 32 dpv. (c) Group 2 (PDCoV inactivated vaccine immunized samples): fourteen pigs tested positive for neutralizing antibodies in PDCoV at 24 dpv. (d) Group 3 (S-based subunit vaccine immunized samples): four pigs tested positive for neutralizing antibodies in PDCoV at 21 dpv. (e) Group 4 (S subunit vaccine immunized samples): five pigs tested positive for neutralizing antibodies in PDCoV 5 days after birth. Dotted lines represent threshold values for nAb positivity. nAb result of log_2_ ≥ 3 is positive, and log_2_ < 3 is negative.

**Figure S15. ROC curve of the DMI≥2 detection assay.** AUC = 0.944, 95% Clopper-Pearson CI: sensitivity (8/9) = 51.75%–99.72%, specificity (3/3) = 29.24%–100%.


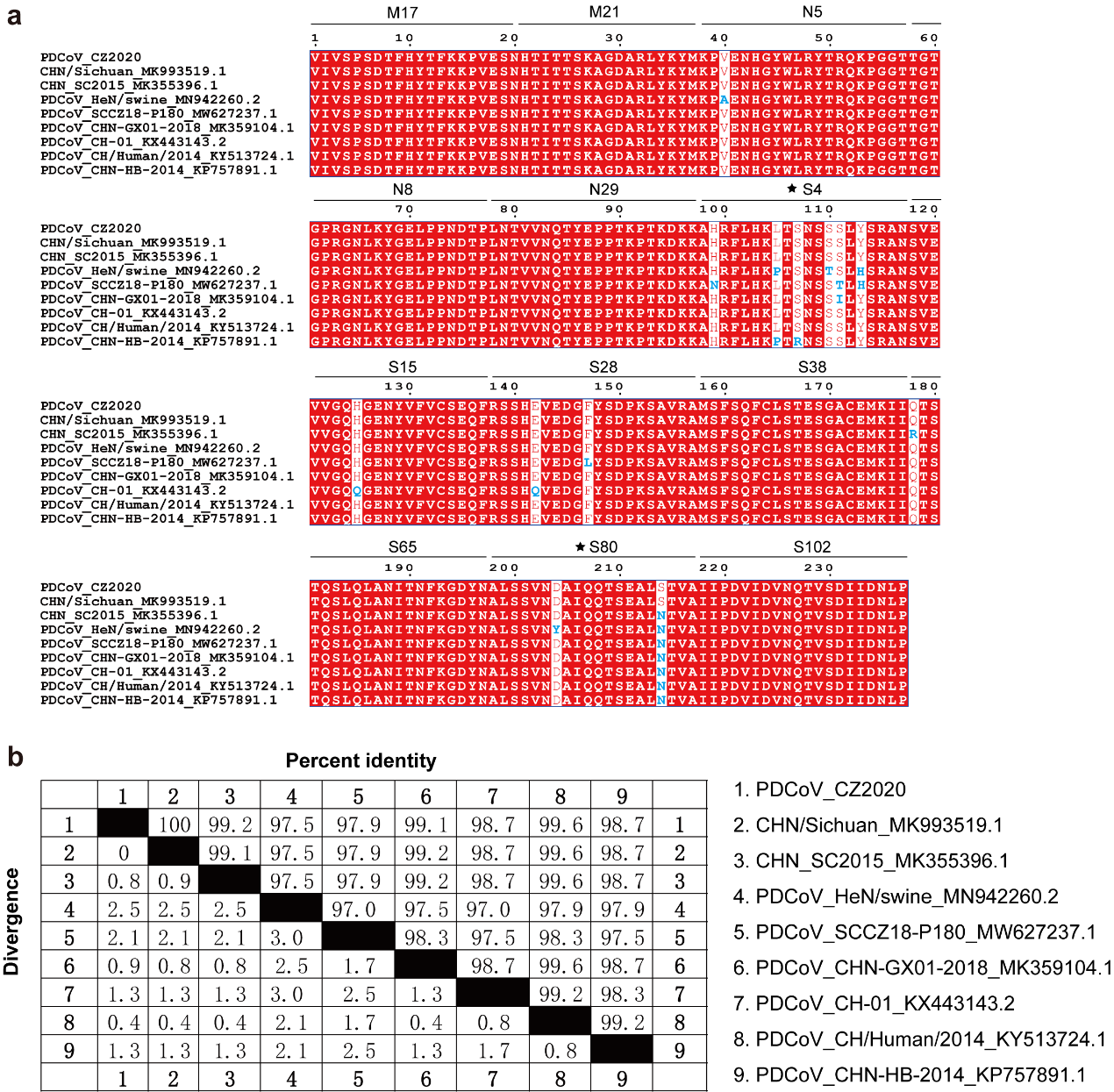


**Figure S16. Sequence conservation analysis of 12 immunodominant peptides among representative PDCoV strains.** (a) Amino acid alignment of 12 selected peptides (M17, M21, N5, N8, N29, S4, S15, S28, S38, S66, S80, S102) across nine representative PDCoV strains, including the reference strain PDCoV_CZ2020. Red shading indicates conserved residues; blue boxes highlight amino acid variations. (b) Matrix showing pairwise percent identity (upper triangle) and divergence (lower triangle) among the 9 PDCoV strains based on the combined sequences of the 12 peptides. Strain numbering corresponds to the right-hand list.


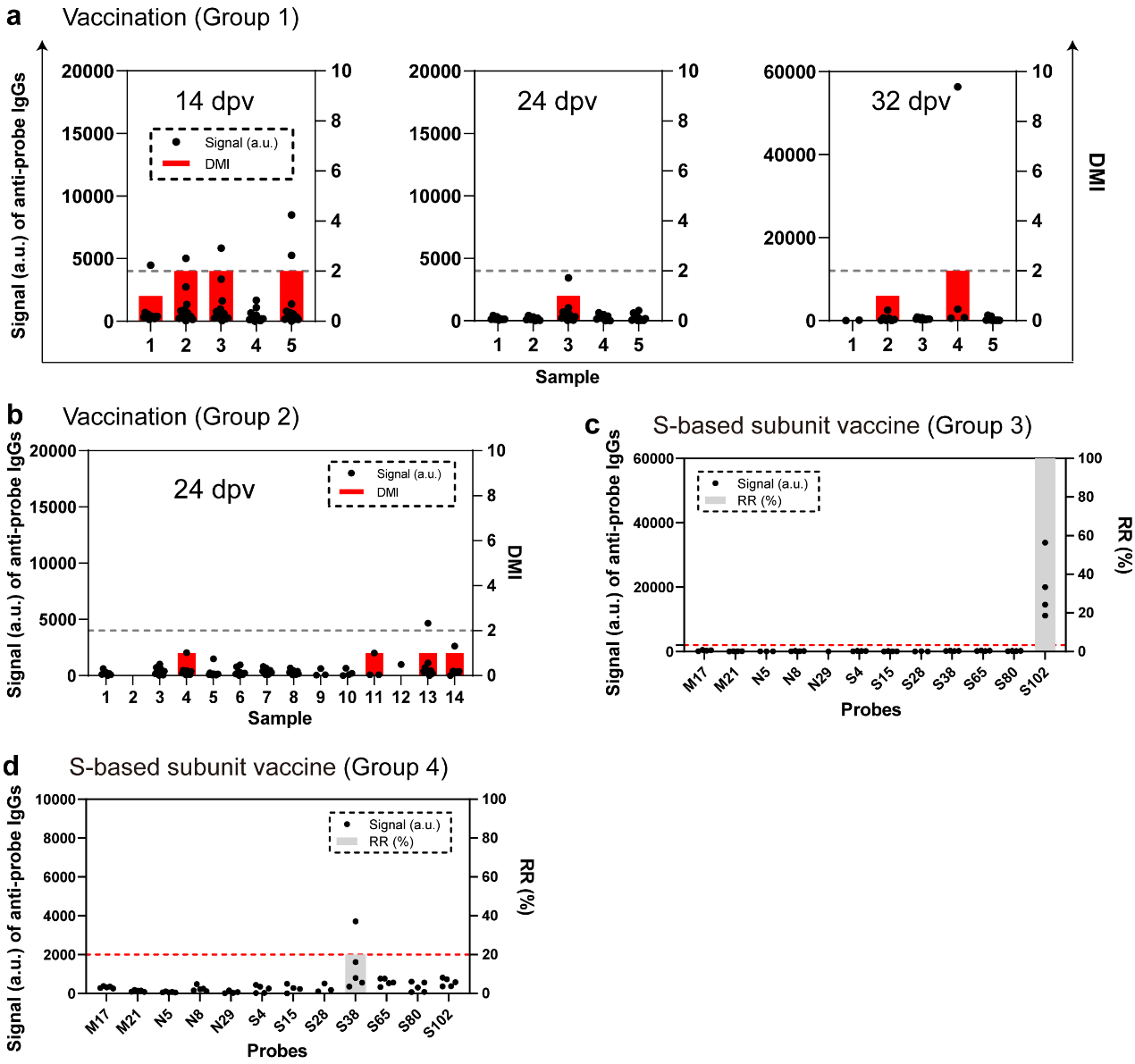


**Figure S17. Statistical analysis of signal and DMI for the group of PDCoV immunized.** (a) Statistical analysis of signal and DMI for five pigs in Group 1 with PDCoV inactivated vaccine immunization at 14 dpv, 24 dpv, and 32 dpv. (b) Statistical analysis of signal and DMI for fourteen pigs in Group 2 with PDCoV inactivated vaccine immunization at 24 dpv. (c) Results of peptide antibody testing for Group 3 with S-based subunit vaccine immunization (n=4). (d) Results of peptide antibody testing for Group 4 with S-based subunit vaccine immunization (n=5). The black dots represent individual signal of anti-peptide IgGs, the red bars indicate DMI, and the gray bars indicate RR percentages. The gray dashed line represents the DMI positivity threshold, and the red dashed line represents the signal positivity threshold.


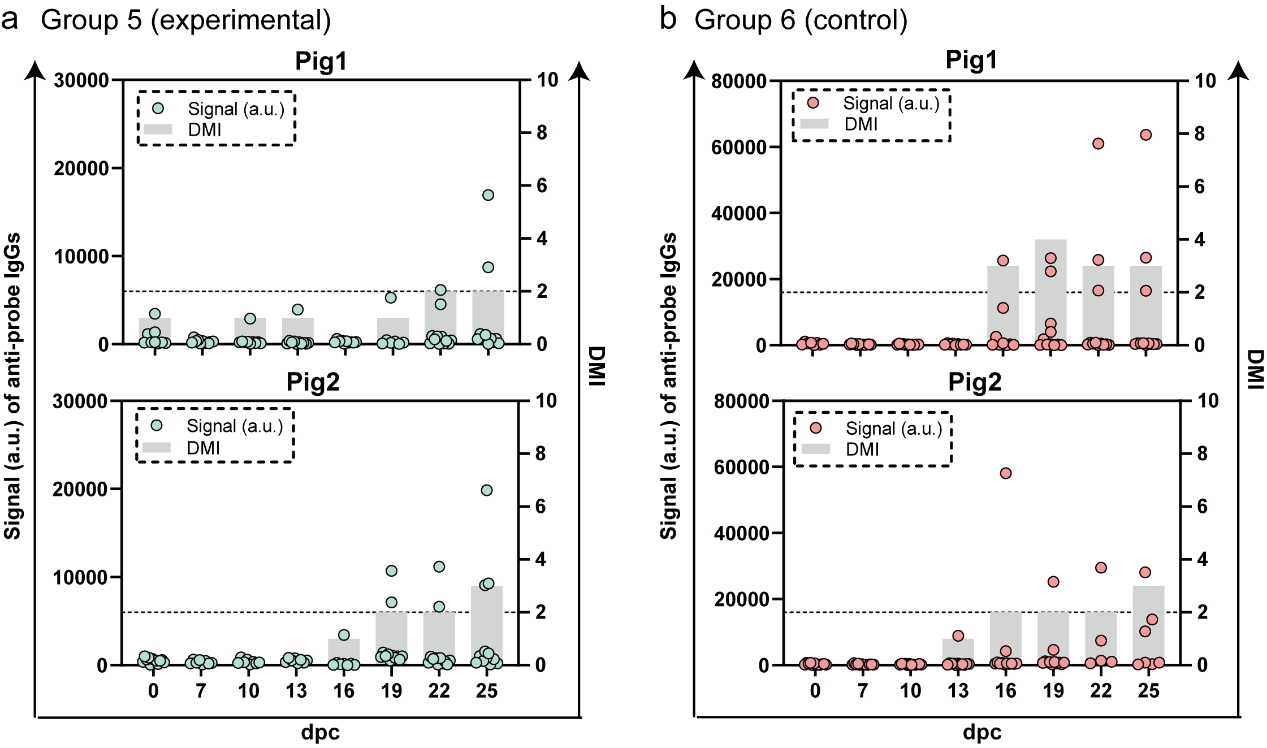


**Figure S18. Comparison the response of anti-probe IgGs between experimental (Group 5) and control (Group 6) pigs after PDCoV challenge.** (a) Signal of anti-probe IgGs (circles) and DMI（grey bars) for two pigs in Group 5 (experimental), which received the PDCoV vaccine inactivated before challenge. Pig1 was tested positive untile 22 dpc, while pig2 was tested positive at 19 dpc. (b) Signal of anti-probe IgG (circles) and DMI (grey bars) for two pigs in Group 6 (control), which were challenged without vaccination. Two pigs were tested positive at 16 dpc.
The dashed line represents the DMI positivity threshold.

Given the wide spread asymptomatic infection of PDCoV in pigs, there is a risk of zoonotic. To demonstrate PPHM_PDCoV_’s utility in public health surveillance, we selected 311 human sera samples from two types of human serum samples across three different regions to check for possible spillover (**Table S5**). The results of PPHM_PDCoV_ showed negative (RR= 0) (**Fig S16a**). Analysis of the response of anti-peptide IgG revealed that the total RR of S65 was 17.7%, other peptides showed no response or only responded once (**Fig S16b-e**), but these were deemed negative due to DMI < 2. These results suggest that, under the current detection method, no PDCoV-specific IgGs were detected in human cohort tested. However, this does not rule out the possibility of low-level or regionally distinct infections.


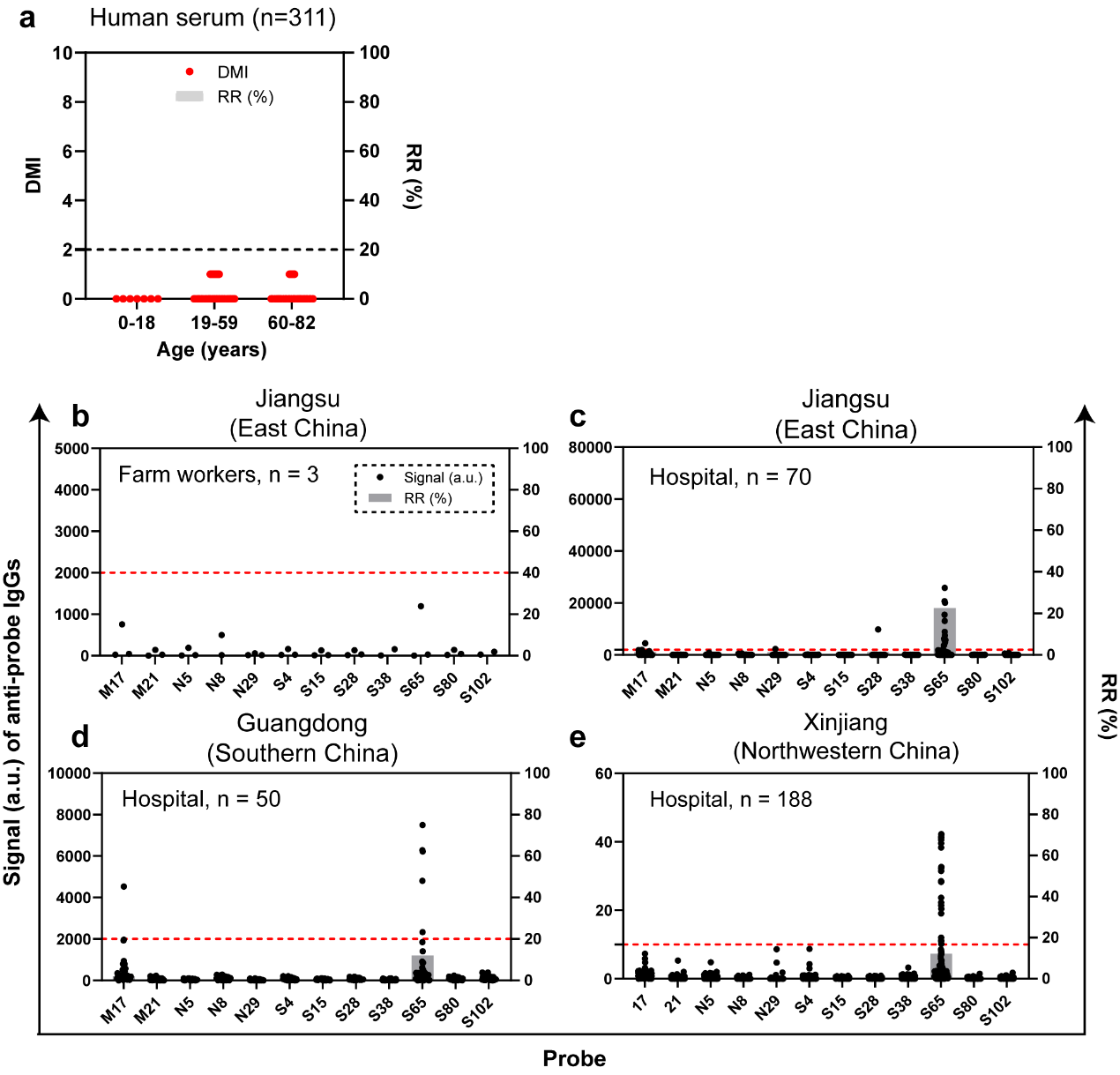


**Figure S19. Results of PPHM_PDCoV_ testing on 311 humans sera samples.** (a) A total of 311 human serum samples were tested using PPHM_PDCoV_. None of the sera samples showed a response. The red dots represent individual DMI values, and the gray bars indicate RR percentages, with a dashed line represents the DMI positivity threshold. The response of 12 PPHM_PDCoV_ probes was analyzed in serum samples from 3 farm workers (b) and 70 humans physical serum samples (c) in jiangsu province. (d) The response of 12 PPHM_PDCoV_ probes was analyzed in serum samples from 50 humans physical serum samples in guangdong province. (e) The response of 12 PPHM_PDCoV_ probes was analyzed in serum samples from 188 humans physical serum samples in xinjiang uygur autonomous region. Black dots represent individual signal, and gray bars indicate RR for each probe. The red dashed line represents the signal positivity threshold.

**Table S1 List of specialized term / abbreviations and definition**

| **Term / Abbreviation** | **Definition** |
| --- | --- |
| Differentiating infected from vaccinated animals (DIVA) | Differentiation of infected from vaccinated animals |
| Digital Microarray Index (DMI) | DMI is the sum of all probe assignments—any probe of PPHM is assigned a response of 1, and no response is assigned a value of 0. |
| IgG sero-dynamics (IsD) | IgG kinetics against responding epitope containing peptides |
| IsD aided epitope discovery (IsDAED) | The IsD curves aided host specific linear peptide identification |
| Non-reproducible interaction (NRI) | SignalRep1/SignalRep2 ≤ 0.5 and SignalRep2 ≥ 2, or SignalRep1/SignalRep2 ≥ 2 and SignalRep1 ≥ 2 ("Rep" is repeat ). |
| Non-specific interaction (NSI) | Interaction between an antibody and a non-cognate epitope, traditionally implies reproducible interactions. |
| Persistently produced IgGs (PPIs) | IgGs that recognize conformational epitopes (i.e., proteins) of antigens. These antibodies are produced during later stages of the immune response and persist for a longer duration, usually over 200 days. PPIs can only be detected by protein-based assays and not by peptide-based methods. |
| Protein-peptide hybrid microarray (PPHM) | A peptide protein hybrid microarray |
| Response rate (RR) | RR = number of positive samples / number of total samples |
| Specific interaction (SI) | Interaction between an antibody and its cognate epitope; traditionally implies reproducible interactions. |
| Transiently produced IgGs (TPIs) | IgGs that recognize linear epitopes (i.e., peptides) of antigens. They typically appear during the early stages of the immune response and have a short duration, usually lasting less than 60 days. TPIs can be detected by both peptide-based and protein-based assays. |

**Table S2 The SI probes of PDCoV**

| **Pig ID** | **Protein** | **Peptide** | | | | **Number of Epitopes** |
| --- | --- | --- | --- | --- | --- | --- |
|  |  | **E** | **M** | **N** | **S** |  |
| Pig1 | S/N | - | M17/21 | N5/8/33 | S4/15/102/114 | 11 |
| Pig2 | S/N | - | M17/21 | N5/9/30/33 | S4/38/65/80/86/89/102/114 | 16 |
| Pig3 | S/N | - | M17/21 | N10/19/29/33 | S4/28/89 | 11 |
| Total | S/N | - | M17/21 | N5/8/9/10/19/29/30/33 | S4/15/28/38/65/80/86/89/102/114 | 22 |

**Table S3 Candidate epitopes for PDCoV**

| **Pig ID** | **Type1** | **Type2** | **Type3** | **Number of Epitopes** |
| --- | --- | --- | --- | --- |
| Pig1 | S/N | M21/N5/N8/S4/S15/S102 | M17 | 9 |
| Pig2 | S/N | M21/S38/S65/S80/S102 | M17 | 8 |
| Pig3 | S/N | M21/N29/S4/S28 | M17 | 7 |
| Total | S/N | M21/N5/N8/N29/S4/S15/S28/S38/S65/S80/S102 | M17 | 14 |

**Table S4 Response rate of PDCoV candidate epitopes in negative serum**

| **Group** | **Probe** | **Response Rate**  **(%)** |
| --- | --- | --- |
| High antigenicity | S | 74 |
| Moderate antigenicity | N | 26 |
| Low antigenicity | M17 | 2 |
|  | M21 | 0 |
|  | N5 | 2 |
|  | N8 | 6 |
|  | N29 | 2 |
|  | S15 | 0 |
|  | S28 | 0 |
|  | S38 | 12 |
|  | S65 | 0 |
|  | S80 | 0 |
|  | S102 | 6 |

**Table S5 The source and distribution of human serum**

| **Province** | Jiangsu (East China) | | Guangdong  (Southern China) | Xinjiang  (Northwestern China) | Total |
| --- | --- | --- | --- | --- | --- |
| Source | Farms | Hospital | Hospital | Hospital |  |
| Number | 3 | 70 | 188 | 50 | 311 |
| **Sex** |  |  |  |  |  |
| Female | 0 (0.0%) | 49 (70.0%) | 50 (26.6%) | 33 (66.0%) | 132 |
| Male | 3 (100%) | 21 (30.0) | 138 (73.4%) | 17 (34.0%) | 179 |
| **Age** |  |  |  |  |  |
| 0-18 | 0 (0.0%) | 2 (2.9%) | 5 (2.7%) | 0 (0.0%) | 7 |
| 19-59 | 3 (100%) | 62 (88.6%) | 148 (78.7%) | 45 (90.0%) | 258 |
| 60-82 | 0 (0.0%) | 6 (8.6%) | 35 (18.6%) | 5 (10.0%) | 46 |

In the present PDCoV study, we selected three challenge serum samples (0 dpc) from experimentally infected pigs as negative controls, and nine serum samples collected between 9~15 dpc from the same animals as positive controls. Sensitivity and specificity were calculated across a range of candidate DMI thresholds. DMI ≥ 2 yielded the highest diagnostic performance, with optimal balance between sensitivity and specificity.

**Table S6. Diagnostic performance of different DMI thresholds**

| **DMI** | **Sensitivity** | **Specificity** | **Youden Index** |
| --- | --- | --- | --- |
| ≥1 | 100% | 0% | 0 |
| ≥2 | 88.90% | 100% | 0.889 |
| ≥3 | 44.40% | 100% | 0.444 |
| ≥4 | 33.30% | 100% | 0.333 |
| ≥5 | 33.30% | 100% | 0.333 |
| ≥6 | 11.10% | 100% | 0.111 |
